# Effect of Mixed Strains on Microbial Community and Flavor Metabolites in Fermentation Process of Chi-Flavor Baijiu

**DOI:** 10.3390/foods13213497

**Published:** 2024-10-31

**Authors:** Puxi Fan, Xuyu Liang, Yongtao Fei, Wenhong Zhao, Jinglong Liang, Weidong Bai, Songgui He

**Affiliations:** 1 Guangdong Provincial Key Laboratory of Lingnan Specialty Food Science and Technology, Zhongkai University of Agriculture and Engineering, Guangzhou 510225, China; fpx20112021@163.com (P.F.); liangxuyu1@zknygchy.onexmail.com (X.L.); zhaowenhong2002@126.com (W.Z.); jinglong_liang@zhku.edu.cn (J.L.); 2 Key Laboratory of Green Processing and Intelligent Manufacturing of Lingnan Specialty Food, Ministry of Agriculture, Zhongkai University of Agriculture and Engineering, Guangzhou 510225, China; 3 Institute of Modern Agricultural Engineering Innovation, Zhongkai University of Agriculture and Engineering, Guangzhou 510225, China; 4 Guangdong Jiujiang Distillery Co., Ltd., Foshan 528203, China; office@kiukiang.com

**Keywords:** chi-flavor baijiu, mix strains, volatile flavors, microbial community

## Abstract

The distinct flavor of chi-flavor baijiu (CFB) has garnered significant attention in China. After the optimization of fermentation conditions, *Pichia anomala* and *Lactobacillus plantarum* were introduced into the fermentation process to enhance the flavor. Samples inoculated with these mixed strains (SY) exhibited higher levels of alcohol (from 33.04 to 178.55 mg/L) and esters (from 49.51 to 130.20 mg/L) compared to the control group (KB). In SY, *P. anomala* and *L. plantarum* were the predominant microorganisms, while *Pediococcus* and *Saccharomyces* were more prevalent in KB. Moreover, 68 volatile flavor compounds were detected in SY, as opposed to 64 in KB. Notably, *Pichia* showed a positive correlation with key flavor compounds. The synergistic fermentation with exogenous strains led to a 52.38% increase in phenethyl alcohol and a 4.91% increase in ethyl lactate. Additionally, the levels of other flavor compounds, like ethyl acetate, γ-nonanolactone, and (E)-2-octenal, also increased. The results demonstrated that the addition of *P. anomala* and *L. plantarum* to the fermentation process of CFB significantly increased the contents of flavor compounds. This research reveals valuable insights into flavor enhancement and the microbial community dynamics in CFB production.

## 1. Introduction

Baijiu, one of China’s earliest solid-state fermented distilled spirits, boasts a history spanning 5000 years [1]. Traditionally, it was yielded through grain fermentation and distillation, with esters and alcohols serving as its primary volatile compounds [2]. Recent research has identified chi-flavor baijiu (CFB) as a distinct aromatic category of baijiu [3]. CFB contains a complex microbial community, with microorganisms responsible for producing most flavor compounds. The composition of these compounds is largely determined by the diversity and dynamics of microbial communities [4]. Consequently, recent studies have focused on understanding the characteristics of microbial communities during the fermentation and their impact on flavor compound composition, as well as attempting to improve the flavor and quality of Baijiu.

Numerous studies have demonstrated that the addition of exogenous and targeted microbial strains can enhance the production of flavor metabolites [5,6,7,8]. Styger and Hirst et al. found that *P. anomala* was able to produce a wide range of flavor substances (alcohols, acids, esters, ketones, hydrocarbons, etc.) and was able to withstand extreme environmental complexity. If fermented under anoxic conditions, *P. anomala* can also activate key enzymes in the fermentation process [9], thus increasing the contents of flavor substances (ethanol, ethyl acetate, etc.) to varying degrees. This increase in flavor contents can be achieved if *P. anomala* is mixed with *Saccharomyces cerevisiae* in the fermentation process [10]. *L. plantarum* is highly acid-resistant. It can survive in different environments. Secondly, it can directly provide nutrients for the growth and metabolism of other microorganisms [11]. Furthermore, *L. plantarum* is able to regulate the acidic environment during the fermentation process, which is conducive to glycation and fermentation. In the late stage of fermentation, the metabolism of *L. plantarum* produces a large amount of lactic acid, which effectively inhibits the growth of some bacteria [12]. Organic acids, such as lactic acid, acetic acid, octanoic acid, and succinic acid, can form various types of esters through esterification, producing compounds like ethyl acetate, ethyl lactate, and ethyl caprylate. These esters not only contribute to the unique flavor of baijiu but also serve as a key source of aromatic substances. 

Ethyl acetate content serves as a standard measure of flavor quality in light-flavor baijiu, with *Pichia pastoris* as a crucial player in its production [13,14]. When compared to the use of individual strains, the incorporation of mixed exogenous strains has been shown to enhance the flavor of CFB in depth. As an illustration, mixed-strain fermentation using *Pichia kudriavzevii* and *Saccharomyces cerevisiae* improved lactic acid tolerance within the mixed culture [15]. Moreover, the co-inoculation of *Saccharomyces cerevisiae* and *Wickerhamomyces anomalus* at specific ratios significantly increased the ethyl acetate content in sauce-flavor baijiu [16]. Several studies have recognized phenylethyl alcohol and (E)-2-octenal as key factors in the overall flavor of CFB, recognizing them as characteristic aromas of CFB [17,18]. To further improve the aroma of CFB, it is crucial to investigate the intricate pertinence between the fermentation process, volatile flavor compounds, and microorganisms. However, previous research on CFB has primarily concentrated on volatile components and unique aromatic compounds [18,19], with limited studies focusing on improving CFB’s flavor through the addition of exogenous strains.

High-throughput sequencing technologies have been extensively utilized to analyze microbial communities in diverse categories of baijiu [18]. Additionally, Headspace Solid-Phase Microextraction Gas Chromatography–Mass Spectrometry (HS-SPME-GC/MS) is a common way of identifying volatile flavor compounds [3,17,20]. In this study, the mixed strains *P. anomala* and *L. plantarum* were introduced into the fermentation process of CFB to investigate their effects on flavor substances and the microbial community. High-throughput sequencing technologies, combined with HS-SPME-GC/MS, were used to evaluate changes in volatile flavors and microbial communities during the CFB fermentation with added mixed strains. This approach enabled a deeper investigation into the mechanisms underlying the formation of these volatile flavors.

## 2. Materials and Methods

### 2.1. Fermentation Characteristics of Exogenously Added Strains

The acid-producing capacity of *L. plantarum* (CICC 24258) was evaluated according to the methodology [21]. The activated *L. plantarum* (1 × 10^7^ CFU/mL) culture was inoculated into 100 mL of MRS agar medium at a 2% (*v*/*v*) concentration and incubated in a biochemical incubator at 30 °C. Samples were periodically collected to measure the total acid content.

Similarly, the ester-producing capacity of *P. anomala* (CICC 1716) was evaluated using the protocol outlined by [8]. The activated *P. anomala* (1 × 10^8^ CFU/mL) culture was inoculated into 150 mL of malt extract agar at a 2% (*v*/*v*) concentration, thoroughly mixed, and then incubated in a biochemical incubator at 30 °C. Samples were periodically collected to determine the total ester content.

In order to assess the ethanol tolerance of both strains, anhydrous ethanol was added at concentrations of 0%, 4%, 8%, 12%, 16%, and 20% (*v*/*v*) to 100 mL of MRS agar medium and malt extract agar, respectively. Each strain was then inoculated at a 2% (*v*/*v*) concentration and incubated in a biochemical incubator at 30 °C for 48 h. Samples were subsequently taken to assess microbial growth, analyzing the ethanol tolerance of the strains.

### 2.2. Optimization of Fermentation Conditions

The fermentation process for CFB was as illustrated in Figure S1. In the process of CFB production, Xiaoqu is usually prepared with rice, rice bran, and wheat bran, which are used as a fermentation starter to provide the mold and yeast necessary for saccharification and alcohol fermentation, respectively [22]. The raw materials of CFB, including rice, soybeans, Xiaoqu, saccharifying enzyme (Aspergillus niger, 200,000 U/mL, Wuxi Boli Bioproducts Co., Ltd., Wuxi, China), and dry yeast, were sourced from Guangdong Jiujiang Distillery Co., Ltd. (Jiujiang, China). The preparation of CFB followed the traditional fermentation method, as described by [23]. First, 2 kg of rice was washed, steamed (the steaming time was 1 h at a temperature of 100–105 °C to ensure that the rice was well pasted), and then cooled to 35 °C. This was then mixed with Xiaoqu (20%), saccharifying enzyme (1%), and dry yeast (2%). Finally, 3 L of distilled water was added to initiate the fermentation, which continued for 15 days.

Optimization of single-fermentation conditions: At the initial stage of CFB fermentation, exogenous additions of *P. anomala* and *L. plantarum* were introduced (Figure S1). Key fermentation parameters, including temperature, pH, fermentation time, and inoculation rate, were adjusted to optimize the production of flavor compounds like total esters, total acids, and alcohol content. To evaluate the impact of these variables, statistical methods, including orthogonal experiments and single-factor experimental designs, were employed. The inoculation rates for both microorganisms were varied from 0% to 12%, in increments of 2%. Additionally, the ideal temperature, pH, and fermentation time were determined using similar experimental approaches. *L. plantarum* and *P. anomala* were activated and inoculated (0%, 2%, 4%, 6%, 8%, 10%, and 12%) into the winemaking bottle, stirred well, sealed, and placed in a constant-temperature biochemical culture incubator at 30° C for continuous cultivation for 15 days. After the fermentation was completed, distillation was performed to measure the alcohol content, total acids, and total esters were measured. These measurements were used to determine the optimal amounts for bacterial strain inoculation *(P. anomala*, 8%; *L. plantarum*, 10%). Next, *P. anomala* and *L. plantarum* were inoculated (optimal inoculum rate) into the winemaking bottle. Only the fermentation temperatures were changed (26 °C, 28 °C, 30 °C, 32 °C, 34 °C, and 36 °C for *P. anomala*, and 20 °C, 24 °C, 28 °C, 32 °C, 36 °C, and 40 °C for *L. plantarum*). Other fermentation conditions remained unchanged. The optimal fermentation temperatures were determined in the same way (*P. anomala*, 30 °C; *L. plantarum*, 36 °C). After determining the optimal fermentation temperature, *P. anomala* and *L. plantarum* were inoculated (optimal inoculum volume and optimal inoculation temperature) in the same way. Only the pH (4, 5, 6, 7, or 8) was changed. Other fermentation conditions remained unchanged. The optimal pH was determined in the same way (*P. anomala*, pH 6; *L. plantarum*, pH 5). Finally, the optimal fermentation time for *L. plantarum* and *P. anomala* was determined by the same method in the case of optimal inoculum rate, fermentation temperature, and pH (*P. anomala* inoculum rate of 8%, fermentation temperature of 30 °C, initial pH of 6, and fermentation time of 4 d; *L. plantarum* inoculum rate of 10%, fermentation temperature of 36 °C, initial pH of 5, and fermentation time of 6 d). On the basis of the single-factor trials, a four-factor, three-level orthogonal design (Tables S1 and S2) was implemented. After thorough analysis, the optimal fermentation conditions for the microorganisms were successfully validated.

Optimization of mixed-fermentation conditions: To determine the optimal levels of key factors—microbial proportion, total inoculation rate (*P. anomala*: *L. plantarum*), pH, and fermentation temperature—a four-factor, three-level orthogonal experiment was conducted (Table S3). The primary evaluation criterion was the overall ester content, in an effort to identify the optimal mixed-fermentation conditions for the dual microbial consortium. In the orthogonal experiment (Table S3), the method of determining the best factor was the same as described above.

### 2.3. Sample Preparation and Analysis of Physicochemical Properties

The optimal conditions for fermenting CFB with *P. anomala* and *L. plantarum* were identified as follows: pH 7, fermentation temperature of 32 °C, an inoculation ratio of 7:3, and an inoculum rate of 10%. *P. anomala* was introduced on the fourth day of fermentation, while *L. plantarum* was added on the sixth day. Samples were collected at specific intervals from 0.5 to 15 days and preserved at −80 °C for future analysis. Control groups were also prepared without the addition of exogenous strains, with all other fermentation conditions kept constant. The collected samples were analyzed for total esters, total acidity, and alcohol content [24,25]. The overall ester content was measured as per the official Chinese standard method [26]. Total acidity (titratable acidity) was measured by titrating the samples to a pH of 8.2 using 0.1 M NaOH. The pH was determined with a digital pH meter (STARTER 3100, Ohaus, Shanghai, China) [27]. The alcohol content was gauged through gas chromatography.

After centrifugation, 1 mL of the supernatant was extracted, diluted to a concentration of 10^−2^, and mixed with 100 μL of 2% acetic acid solution before analysis. The alcohol content was gauged through gas chromatography (model 7820A; Agilent, Agilent Technologies Shanghai Analytical Instruments Co., Ltd., Shanghai, China) with an LZP-950 column (50 m × 0.32 mm × 1 μm; Agilent) and flame ionization detector (FID) [28,29]. The flows of helium (carrier gas), hydrogen, and air were 1 mL/min, and 35 mL/min, and 350 mL/min, respectively. The injector and detector temperatures were set at 230 °C. A sample volume of 10 μL was injected with a split proportion of 30:1. The oven temperature was programmed as follows: initially set to 60 °C for 0.1 min, then raised at a rate of 2 °C/min to 70 °C, where it was kept for 1 min. This was followed by a rise at 5 °C/min to 80 °C, and subsequently at 11 °C/min to 110 °C and held for 18 min. Lastly, the temperature was raised at 8 °C/min to 250 °C and kept there for 10 min.

Following centrifugation, the supernatant was also dissected for organic acids through high-performance liquid chromatography (HPLC) [30]. Chromatographic separation was achieved using a Zorbax SB-Aq column (4.6 × 250 mm, 5 μm; Agilent Technologies Shanghai Analytical Instruments Co., Ltd., Shanghai, China) at a column temperature of 30 °C. UV detection was performed at a wavelength of 214 nm with a flow rate of 0.8 mL/min. The mobile phase consisted of methanol (A) and a 0.01 mol/L KH_2_PO_4_ buffer solution (pH 2.85, B) at a ratio of 3:97. A 10 μL sample volume was injected, and the total analysis time was 40 min.

### 2.4. Volatile Component Analysis

Complying with the approach reported by [31], volatile flavor components of CFB were analyzed using HS-SPME-GC/MS technology (6890-5973, Agilent Technologies, Inc., Beijing, China). In brief, 14 mL samples of centrifuged fermentation broth were blended with 4 g of NaCl and 200 μL of an internal standard (2-octanol, 1 mg/L, Aladdin Industrial Corporation, Shanghai, China) and then sterilized. A 50/30 μm divinylbenzene/carboxen/polydimethylsiloxane (DVB/CAR/PDMS)-coated fiber (Supelco, Inc., Bellefonte, PA, USA) was introduced for headspace extraction at 50 °C for 10 min. Volatile fragrance compounds were dissected using an Agilent 8890-5977B GC-MS (Agilent Technologies, Inc., Beijing, China) with a DB-WAX UI capillary column (30 m × 0.25 mm × 0.25 μm; Agilent Technologies, Inc., Beijing, China). The oven temperature program was initiated at 40 °C for 6 min, increased to 100 °C at a rate of 5 °C/min and kept there for 6 min, and ultimately ramped to 150 °C at 6 °C/min for 8 min. The flow rate of the carrier gas (helium) was set at 1 mL/min. The temperatures for the MS interface, MS source, and MS quadrupole reached 250 °C, 230 °C, and 150 °C, respectively. The MS ran in an electron impact pattern with an electron energy of 70 eV and a scanning range of 47–550 *m*/*z*. Volatiles were characterized and analyzed using the National Institute of Standards and Technology (NIST) spectral library. The retention index (RI) of the substances was calculated based on the retention times of n-alkanes C_7_–C_40_ (49452-U, Sigma-Aldrich, Shanghai, China) detected under the same chromatographic conditions [32] and the formula for RI was as follows:

$$RI=100*(Z+\frac{T_{x}-T_{z}}{T_{Z+1}-T_{Z}})$$

where Z is the carbon number of the n-alkane adjacent to (before) the target peaks. $T_{x}$, $T_{z}$, and $T_{Z+1}$ are the target compound retention time, retention time of n-alkanes adjacent to (before) the target peak, and retention time of n-alkanes adjacent to (after) the target peak, respectively.

The concentration of each volatile compound was calculated using the internal standard method with the following formula:

$$Concentration\;of\;flavor\;compound=\frac{C*F_{1}}{F_{2}}$$

where C is the ratio of the peak of the flavor compound to the peak area of the 2-octanol. F_1_ and F_2_ are the quality of the 2-octanol and the sample, respectively.

To determine the effects of various flavor compounds on the overall sensory profile of CFB, the primary flavor components during CFB fermentation were studied. Consequently, further analysis of the odor activity value (OAV) of various flavor compounds during CFB fermentation was required. The OAV is the ratio of the concentration of a flavor compound to its threshold.

### 2.5. DNA Extraction and High-Throughput Sequencing

The total DNA from bacteria and fungi was obtained from the sample collected through the FastDNA^®^SpinKitforSoil (MP Biomedicals, Santa Ana, CA, USA), following the manufacturer’s guidelines. The extracted DNA served as templates for amplifying bacterial 16S rRNA genes and the ITS1 areas of fungal ITS rRNA genes. Bacterial amplification used primers 27F (AGRGTTTGATYNTGGCTCAG) and 1492R (TASGGHTACCTTGTTASGACTT), while fungal amplification utilized primers ITS1 (F-CTTGGTCATTTAGAGGAAGTAA) and ITS4 (R-TCCTCCGCTTATTGATATGC). High-quality sequences were employed to analyze operational taxonomic units (OTUs), figured out fungal alpha diversity indices, and assess species abundance. The Correlation Calculator and Cytoscape software 3.9.0 were adopted to rate the correlations between the main flavor compounds and microbial communities.

### 2.6. Statistical Analysis

Chemical composition and flavor analyses for each sample were executed in triplicate. Statistical analysis was implemented through IBM SPSS Statistics 25 (SPSS Inc. Chicago, IL, USA) through analysis of variance (ANOVA). Chemical index data were processed with Origin version 8.0 (OriginLab Inc., Hampton, MS, USA) and Excel 2019 (Microsoft Corp., Redmond, WA, USA). Principal Component Analysis (PCA) was employed to investigate the relationships between volatile flavor compounds and microorganisms.

## 3. Results and Discussion

### 3.1. Fermentation Characteristics of Exogenous Strains

**Figure 1:**
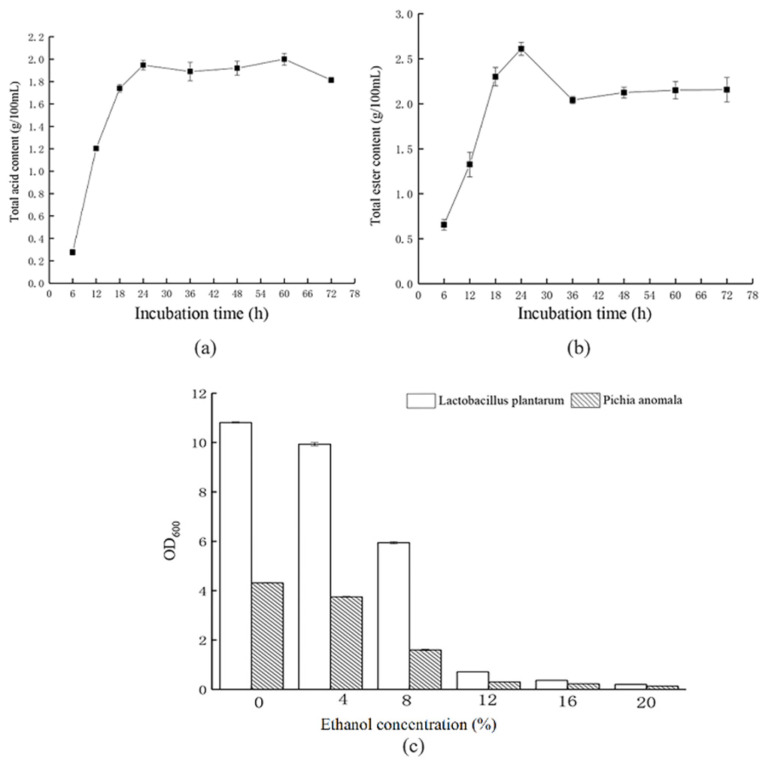
Fermentation characterization of *L. plantarum* and *P. anomala*: acid-producing capacity of *L. plantarum* (**a**), ester-producing capacity of *P. anomala* (**b**), and the capacity of the two strains to tolerate ethanol (**c**).

*L. plantarum* demonstrated a rapid increase in acid production in MRS medium, rising from 0.25 g/100 mL at 6 h to 1.95 g/100 mL at 24 h. After 24 h of fermentation, the total acid content remained relatively stable, showing minimal fluctuations (Figure 1a). The trend in acid production by *L. plantarum* closely mirrored its growth pattern, primarily because lactic acid is a key metabolite for lactic acid microbes [33]. In the fermentation process, the production of organic acids, including lactic acid and acetic acid, by *L. plantarum* enhanced the flavor and taste of baijiu. Additionally, it provided ample precursors for the composition of ester compounds [34].

*P. anomala* exhibited a notable increase in total ester content in PDA medium from 6 h to 24 h, reaching a maximum of 2.61 g/100 mL at 24 h, highlighting its strong ester-producing capability (Figure 1b). The ester content gradually decreased from 24 h to 36 h and stabilized thereafter. The decrease in ester content might have been because of hydrolysis of esters by lipase from *P. anomala* [35]. Ester compounds play a significant role in contributing to the characteristic flavor profile of CFB [8,30].

The tolerance of *L. plantarum* and *P. anomala* to various ethanol concentrations was as depicted in Figure 1c. After 48 h of cultivation, both *L. plantarum* and *P. anomala* showed robust growth in ethanol solutions with concentrations of up to 8%. Although growth was inhibited at an ethanol concentration of 12%, there was still some increase in cell density. During the brewing process of CFB, the maximum alcohol concentration in the mash reached 12%vol [31], indicating that both *L. plantarum* and *P. anomala* can tolerate this level of alcohol. This tolerance allows for continued growth metabolism and the accumulation of additional flavor compounds, thereby enhancing the quality of the baijiu.

### 3.2. Optimization of Fermentation Conditions for Strain-Enhanced CFB

The optimal fermentation conditions for single-strain fermentation of *P. anomala* were as follows: a pH of 7, an inoculum size of 6%, a fermentation temperature of 32 °C, and inoculation on the 5th day. Under these conditions, the alcohol content of the distilled CFB reached 32.47% vol, with a total ester content of 0.707 g/L. For single-strain fermentation of *L. plantarum*, the optimal conditions were as follows: pH of 5, an inoculum size of 12%, a fermentation temperature of 36 °C, and inoculation on the 6th day. Validation experiments determined that the average alcohol content for this optimal combination was 29.60% vol, with an average total ester content of 0.629 g/L. The overall ester content in SY was nearly three times higher (0.194 g/L) compared to the blank control, indicating that mixed fermentation significantly improved the total ester content of the CFB.

The optimal conditions for mixed fermentation of CFB were as follows: a mixed-culture fermentation ratio of 7:3 (*P. anomala*: *L. plantarum*), an inoculation rate of 10%, a pH of 7, and a fermentation temperature of 32 °C. Under these circumstances, the average alcohol content of the distilled CFB was 32.44% vol, with an overall acidity of 0.221 g/L and an overall ester content of 0.852 g/L. Compared to CFB fermented solely with *P. anomala* (0.707 g/L) or *L. plantarum* (0.629 g/L), mixed fermentation demonstrated superior results, significantly increasing the total ester content of the CFB.

### 3.3. Organic Acid Analysis by HPLC

The total organic acid content was analyzed using HPLC, revealing a significant increase in the total organic acid levels in SY and KB samples during the first two days of fermentation (*p* < 0.05). This initial surge was primarily due to the high nutrient content in the mash, which promoted the rapid growth of acid-producing microbes (Table 1). During the mid-fermentation period (days 2 to 8), the rate of increase in organic acid content for both SY and KB samples slowed. This deceleration can be ascribed to the increased alcohol concentration and decreased pH, which inhibited the growth and metabolism of certain acid-producing microorganisms. From days 5 to 8, fluctuations in total organic acid content were observed, mainly due to the introduction of exogenous strains (*P. anomala* on day 5 and *L. plantarum* on day 8). In the late fermentation stage (days 9 to 15), the organic acid content in SY and KB samples exhibited an upward trend. At the end of fermentation, SY had an overall organic acid content of 8.43 g/L, significantly higher than the 7.58 g/L observed in KB (*p* < 0.05). This increase indicates that the addition of exogenous strains, especially the *L. plantarum* with its strong ability to produce lactic acid, enhanced the organic acid content in the later fermentation stages, promoting ester synthesis and thereby enhancing the stability and quality of the baijiu [36,37].

The organic acids in the SY and KB samples were analyzed, focusing on acetic acid, malic acid, succinic acid, and lactic acid. These acids accounted for 86.83% and 79.82% of the total organic acids in the SY and KB samples, respectively. This indicates that lactic acid, succinic acid, acetic acid, and malic acid, with their higher concentrations, were the predominant organic acids in the CFB. Together, they contributed significantly to the primary acidic flavor compounds, further promoting the production of esters, which was in accordance with the findings of our previous study [31]. In particular, the acetic acid concentration in the KB sample was 1.402 g/L, notably higher than the 1.212 g/L observed in the SY sample at the end of fermentation (Table 1). The malic acid content rose throughout fermentation in both groups, reaching 1.586 g/L in SY and 1.374 g/L in KB (Table 1). Additionally, the succinic acid content in both samples gradually increased during fermentation, with the SY sample showing a higher concentration of 1.855 g/L compared to 1.642 g/L in the KB sample at the end of fermentation.

During the early fermentation stages, the lactic acid content rose quickly in both groups (Table 1). This rise was due to the conversion of pyruvic acid from glycolysis into lactic acid by lactic dehydrogenase in lactic acid bacteria, with such genera as *Lactobacillus*, *Pediococcus*, and *Weissella* [38]. At the end of fermentation, the lactic acid content in the SY and KB samples was 2.081 g/L and 2.166 g/L, respectively. On day 7, the decrease in lactic acid content in the SY samples corresponded to a remarkable rise in ethyl lactate content. This suggests that the addition of *L. plantarum* to the CFB culture promoted the formation of ethyl lactate, which, in turn, led to a reduction in lactic acid content. Consequently, the mixed-culture approach enhanced the production of malic and succinic acids, resulting in a more diverse and fruity aroma, enriched taste, and a more balanced aroma profile in the spirit. Although mixed-culture fermentation reduced both acetic acid and lactic acid contents, there was a remarkable rise in ethyl lactate content among the SY samples.

### 3.4. Analysis of Volatile Flavor Substances in Fermentation Processes

**Figure 2:**
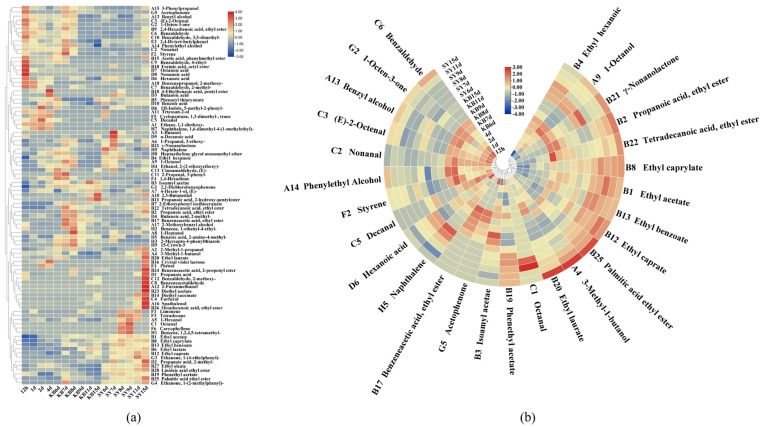
Heatmap of volatile flavors (**a**) and volatile flavors with OAV > 1 (**b**) during CFB fermentation.

Volatile flavor substances in SY and KB were dissected through HS-SPME-GC/MS (Figure 2a). In total, 76 different volatile flavor substances were examined across both samples. Specifically, 68 volatile flavors were identified in the SY samples, comprising 25 esters, 14 alcohols, 9 acids, and 20 other flavor substances. In contrast, 64 flavors were identified among the KB samples, encompassing 22 esters, 13 alcohols, 9 aldehydes, 7 acids, and 13 other flavor substances. Of these, 56 volatile flavors were common to both groups. At the end of fermentation, the overall content of volatile flavor substances reached 337.50 mg/L in SY and 101.04 mg/L in KB. Esters, alcohols, and aldehydes, which together accounted for over 95% of the overall volatile flavor substances (in Figure 2a, the deeper the red color, the higher the content, while the deeper the blue color, the lower the content), were the predominant components in both groups. This finding was in accordance with previous studies that found that esters, alcohols, and aldehydes were the main flavors in chi-flavor and rice-flavor baijiu [39,40].

Among these, the ester content in SY samples was 137.20 mg/L, significantly higher than the 49.51 mg/L found in KB at the end of fermentation. Esters can contribute fruity and floral aromas to baijiu [41]. Additionally, the alcohol content in SY samples was 178.55 mg/L, markedly higher than the 33.04 mg/L in KB samples at the end of fermentation. While alcohols primarily enhance the ester aroma, and some can exhibit floral notes, excessively high alcohol levels may lead to alcohol poisoning and brain paralysis [42]. Furthermore, the aldehyde content in SY samples was 9.12 mg/L, significantly surpassing the 1.44 mg/L in KB samples at the end of fermentation. Aldehydes can carry aromatic components from the baijiu and intensify the aroma release [43]. Consequently, it was evident that the flavor profile of SY was superior to that of KB, suggesting that the introduction of mixed strains may increase the concentration of CFB flavor compounds, particularly esters and alcohols.

It is widely known that compounds with an OAV of 1 or higher contribute significantly to the overall flavor, with a higher OAV contributing more to the flavor profile of baijiu [44]. At the end of fermentation, 21 flavor compounds with OAVs of 1 or greater were identified, including ethyl acetate (B1), phenylethyl alcohol (A14), 1-octen-3-one (G2), ethyl caprylate (B8), and (E)-2-octenal (C3) (Figure 2b). Specifically, SY had 21 substances with OAVs greater than 1, comprising 12 esters, 4 alcohols, 2 aldehydes, and 2 other compounds. The top three flavor compounds by OAV were 1-octen-3-one (G2) (mushroom aroma), (E)-2-octenal (C3) (citrus aroma), and ethyl caprylate (B8) (brandy-like aroma). In comparison, KB had 17 substances with OAVs greater than 1, including 10 esters, 2 alcohols, 2 aldehydes, 1 ketone, and 2 other compounds, with the top three flavor compounds matching those of SY. Additionally, the only lactone flavor identified in this study was γ-nonanolactone, which enhances mouthfeel and provides a coconut scent with notable aroma stability [45].

At the end of fermentation, the concentration of (E)-2-octenal in SY was 0.7482 mg/L, nearly four times higher than in KB. This indicates that the mixed fermentation of CFB produces richer flavors and a more complex mouthfeel. Additionally, the ethyl acetate concentration in SY samples reached 34.81 mg/L, compared to just 13.50 mg/L in KB samples. The ethyl lactate content in SY was 15.86 mg/L, significantly higher than the 10.95 mg/L found in KB. These results demonstrate that mixed fermentation enhances the levels of ethyl acetate and ethyl lactate in CFB. Furthermore, the quality of CFB was assessed based on the concentrations of phenylethyl alcohol and diethyl succinate [46]. The phenylethyl alcohol concentration in SY was 65.74 mg/L at the end of fermentation, while it was only 13.26 mg/L in KB. Diethyl succinate (DES) was detected only in SY, at a concentration of 0.4062 mg/L. DES may enhance the wine’s flavor profile by imparting fruity and sweet undertones [43,47]. Studies have suggested that DES also exhibits anti-inflammatory properties in cellular models of chronic neuroinflammation, indicating its potential as a novel treatment for this condition [48]. The γ-nonanolactone content in SY was higher than in KB, suggesting that the combined fermentation of CFB can produce more complex and enduring aromas, thereby improving the aroma profile of baijiu.

The mixed fermentation of CFB clearly resulted in the production of DES, which further enhanced the richness and uniqueness of SY compared to KB. The PCA plot showed that SY and KB were primarily located on opposite sides, with no overlap, indicating substantial differences in characteristic volatile aromatic compounds like phenylethyl alcohol and ethyl acetate (Figure S3a). The OPLS-DA model revealed significant discrepancies in the levels of 13 volatile aromatic compounds between both groups. These compounds included 2-methyl-1-propanol (A2), 3-methyl-1-butanol (A4), benzyl alcohol (A13), phenylethyl alcohol (A14), ethyl acetate (B1), phenacyl thiocyanate (B5), ethyl lactate (B6), 2-ethoxyphenyl isothiocyanate (B7), diethyl succinate (B14), palmitic acid ethyl ester (B25), ethyl oleate (B27), benzaldehyde (C6), and 2,2-dichlorobenzophenone (G1) (Figure S3c). Thus, the addition of mixed strains significantly increased the levels of characteristic volatile flavor compounds, including phenylethyl alcohol, ethyl acetate, ethyl lactate, and diethyl succinate, thereby enhancing the flavor profiles of CFB.

### 3.5. Changes in Microbial Community During Fermentation

**Figure 3:**
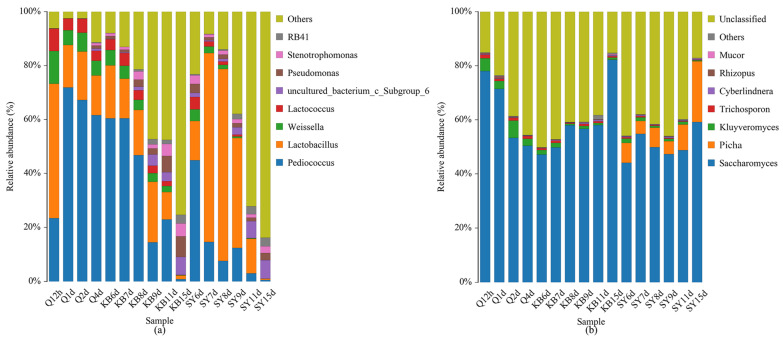
Relative abundances of bacterial (**a**) and fungal (**b**) flora at the genus and species levels during fermentation of CFB.

The 16S rRNA gene sequencing results (Figure 3a) revealed that the predominant bacterial genera in the early fermentation stage were *Pediococcus pentosaceus*, *Lactobacillus fermentum*, *Weissella paramesenteroides*, and *Lactococcus lactis*. Among these, *Pediococcus pentosaceus* was the most abundant, averaging 56% of comparative abundance during the early fermentation stage. Following the addition of *L. plantarum*, there was an obvious increase in the comparative abundance of *Lactobacillus* in the SY sample on day 7. Furthermore, the comparative abundance of *Lactobacillus* remained more elevated among the SY samples compared to the KB samples between days 7 and 11, leading to significant differences in aromatic compounds and organic acids between the SY and KB samples (Figure 2a,b and Figure S2). During the late fermentation stage (days 9 to 15), the relative abundances of *Pediococcus pentosaceus* and *Lactobacillus* continued to decrease. By the end of fermentation, the microbial composition and abundance in both samples were similar, suggesting that the exogenously added *L. plantarum* was suppressed by disadvantageous conditions such as high alcohol concentration, nutrient deficiency, and harmful metabolites during the late fermentation stage.

**Figure 4:**
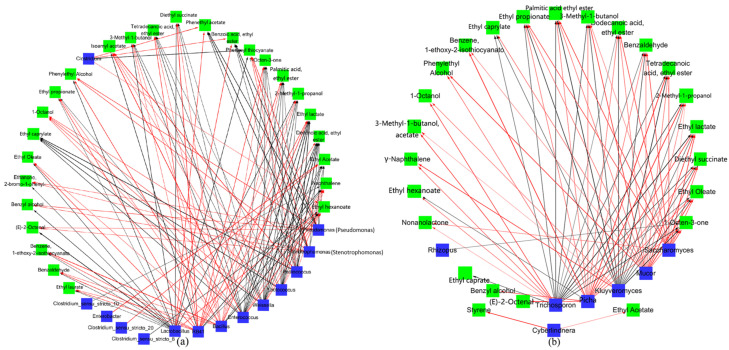
Correlations between microorganisms (bacterial (**a**) and fungal (**b**)) and volatile flavor substances, with significant differences based on Pearson correlation analysis. The red lines represent positive correlations, the black lines represent negative correlations, and the thickness of the lines indicates the strength of the correlation.

The high-throughput sequencing results of fungal ITS genes (Figure 4b) revealed that seven fungal genera were identified in both groups, including *Saccharomyces*, *Pichia*, *Kluyveromyces*, *Trichosporon*, *Cyberlindnera*, *Rhizopus*, and *Mucor*. While seven fungal genera were identified among SY samples, the *Pichia* genus was not found in KB samples. During the early fermentation stages, the abundance of *Saccharomyces* gradually declined, yet it remained the dominant genus, with an average abundance of 63.29%. After the addition of *P. anomala* on day 6, the dominant fungal genera in SY samples were *Saccharomyces*, *Pichia*, and *Kluyveromyces*, whereas KB samples only had *Saccharomyces* and *Kluyveromyces* as dominant genera. The comparative abundance of *Pichia* among SY samples rose prominently from 7.38% on day 6 to a maximum of 22.46% at the end of fermentation. *P. anomala* demonstrated good adaptability to the late fermentation environment and exhibited a competitive survival advantage. Compared to KB samples, the contents of phenylethyl alcohol and esters in SY samples increased by 2.8- and 5-fold, respectively. Previous studies [49,50] have shown that genera such as *Saccharomyces*, *Pichia*, *Kluyveromyces*, and *Trichosporon* produce flavor metabolites such as aldehydes, alcohols, and esters. Therefore, it was speculated that *P. anomala* enhanced the production of esters and phenylethyl alcohol in CFB.

### 3.6. Correlation Analysis Between Volatile Flavors and Communities (Bacterial and Fungi)

The genera *Bacillus* and *Pseudomonas* exhibited a positive correlation with 2-methyl-1-propanol, 3-methyl-1-butanol, and phenylethyl alcohol (Figure 4a). Prior studies have highlighted a significant positive relationship between these genera and certain higher alcohols [14,51,52]. These findings suggest that bacteria within these genera may have the capability to produce higher alcohols.

Regarding ester compounds, consistent microbial effects were examined between the control and experimental groups. *Enterobacter* was found to be strongly positively correlated with ethyl acetate, while *Pediococcus* was negatively correlated with ethyl lactate. *Bacillus* demonstrated positive correlations with most ester compounds. Notably, diethyl succinate showed significant correlations with both SY and KB, indicating that the *Bacillus* genus could enhance the production of various ester compounds, particularly those contributing to characteristic flavors during the fermentation process of CFB. Numerous studies have revealed that *Bacillus* species, like *Bacillus licheniformis* and *Bacillus velezensis*, can secrete esterases to synthesize ethyl esters from short-chain fatty acids [53,54]. Such enzymatic activity increases the concentration of esters, including ethyl acetate, ethyl hexanoate, and ethyl butyrate, in baijiu.

*Mucor* exhibited positive correlations with 2-methyl-1-propanol, 3-methyl-1-butanol, and phenylethyl alcohol, with correlation coefficients of 0.608, 0.788, and 0.801, respectively (Figure 4b). Our research revealed that the phenylethyl alcohol content in SY was approximately five times as high as that in KB by the end of fermentation. *Mucor* has been reported to produce substantial amounts of protease, which converts L-phenylalanine into phenylethyl alcohol [55,56]. To give an example, high ethanol production by *Mucor* was considered to be a key characteristic of this strain during fermentation with starch as the substrate—a finding consistent with this study, where *Mucor* showed a strong correlation with ethanol [57].

Additionally, phenylethyl alcohol, 3-methyl-1-butanol, 2-methyl-1-propanol, and benzyl alcohol all demonstrated strong positive correlations with *Pichia* [58], with correlation coefficients of 0.938, 0.905, 0.858 and 0.847, respectively. *Pichia* has been reported to enhance the synthesis of higher alcohols, particularly increasing the content of phenylethyl alcohol. Regarding esters, *Pichia* showed a strong correlation with ethyl lactate, with a coefficient of 0.765. Moreover, this genus also had a strong correlation with higher fatty acid ethyl esters like ethyl oleate, ethyl propionate, ethyl laurate, diethyl succinate, and palmitic acid ethyl ester, with correlation coefficients of 0.99, 0.975, 0.967, 0.95, and 0.937, respectively. Consequently, both *Mucor* and *Pichia* were key players in forming characteristic volatile flavors in CFB.

**Figure 5:**
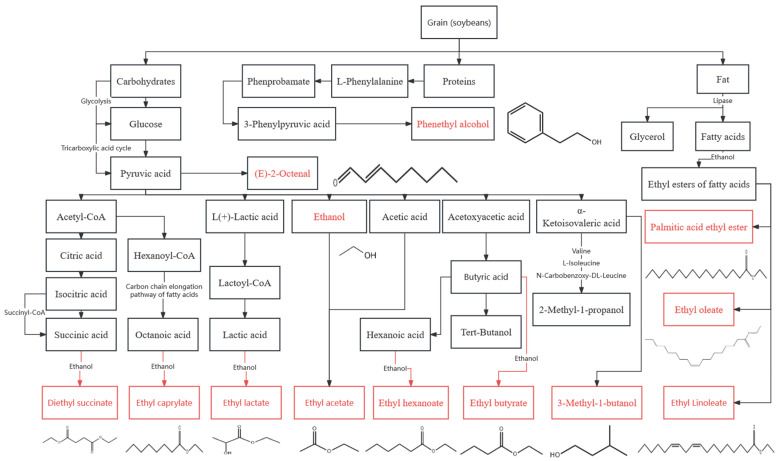
Metabolic networks of selected flavor chemicals during CFB fermentation.

Aromatic compounds exhibited fluctuations during the CFB fermentation. The significant positive relationship between microorganisms and aromatic compounds demonstrated the substantial role that microorganisms play in synthesizing these flavor chemicals (Figure 5). Microbial metabolism facilitated by enzymes in associated metabolic pathways [59] essentially involves a dynamic balance between the synthesis and degradation of specific flavor compounds. To better modulate the metabolism of aromatic chemicals through microorganisms in subsequent studies, the metabolic synthesis pathways of these compounds were summarized. During the CFB fermentation process, microorganisms utilize substrates like proteins, fats, and carbohydrates from soybeans. Carbohydrates were changed into the pivotal metabolic intermediate pyruvate via glycolysis and the tricarboxylic acid cycle (TCA) pathway. Pyruvate was produced via glycolysis. Then, pyruvate was converted to ethanol by pyruvate decarboxylase and alcohol dehydrogenase (Figure 5).

Furthermore, pyruvate can be changed to acetyl-CoA by 2-oxoacid oxidoreductase [59,60]. Then, acetyl-CoA can be further transformed into citric acid, isocitric acid, and succinic acid, which eventually combine with ethanol to form diethyl succinate (DES). Additionally, pyruvate was converted into hexanoyl-CoA and acetoxyacetic acid [60], producing precursors for flavored ethyl esters (hexanoic and octanoic acids) through the fatty acid carbon chain elongation pathway (Figure 5). These acids were subsequently esterified with alcohols (such as ethanol) to form the corresponding ester compounds.

Proteases can hydrolyze soybean proteins to yield amino acids, including L-phenylalanine. These amino acids can then be changed to 3-phenylpyruvic acid by amino acid transaminase and eventually produce phenethyl alcohol. Additionally, some amino acids (e.g., valine, L-isoleucine, and N-carbobenzoxy-DL-leucine) participate in the synthesis of higher alcohols (e.g., 2-methyl-1-propanol and 3-methyl-1-butanol) [43]. The fats in soybeans can also be hydrolyzed to produce fatty acids and glycerol. These fatty acids combine with ethanol to yield ethyl esters of higher fatty acids, including palmitic acid ethyl ester, ethyl oleate, and ethyl linoleate (Figure 5) [61].

Studies on enzymes involved in esterification reactions in baijiu microorganisms have laid a theoretical foundation for these reactions, adopting alcohols and acids as substrates [36]. This was a crucial metabolic pathway for ester formation in CFB [62]. Consequently, the metabolic activities of microorganisms significantly affect the synthesis of flavor compounds during CFB fermentation. Microorganisms metabolize carbohydrates, proteins, and fats in soybeans to produce a diverse array of flavor compounds. Therefore, further research on the metabolic pathways of these microorganisms and the management of corresponding enzyme activities is feasible. Such research could aid in regulating and controlling the flavor compounds produced during CFB fermentation.

### 3.7. Conclusions

Based on the above, this study investigated the formation of flavor compounds during the CFB fermentation process using GC-FID, HS-SPME-GC/MS, HPLC, and high-throughput sequencing. The results showed that *L. plantarum* contributed greatly to the increase in acid content, while the increase in ester content was also associated with *P. anomala*. *L. plantarum* accumulated precursors for ester production, while *P. anomala* had a strong ester-producing ability. In addition, 68 volatile flavor compounds, including aldehydes, alcohols, hydrocarbons, esters, ketones, acids, and phenols, were identified by HS-SPME-GC/MS analysis. During the CFB fermentation process, the types (alcohols, esters, ketones, and hydrocarbons) and contents of volatile flavor compounds (phenylethyl alcohol, (E)-2-octenal, and diethyl succinate) increased significantly. This increase was closely related to *L. plantarum* and *P. anomala*, which were able to enhance the contents of esters under the premise of increasing the alcohol content, proving that the addition of a mixture of *L. plantarum* and *P. anomala* was able to significantly increase the contents of flavor compounds.

Therefore, this study offers valuable insights into the impact of *P. anomala* and *L. plantarum* on the CFB fermentation process, functioning as a reference for the optimization and advancement of CFB fermentation technologies. Future research will explore the changes in flavor precursor substances and the formation pathways of volatile flavor compounds in CFB, along with their application prospects. In conclusion, this study provides a pathway for the formation of increased volatile flavor compounds in CFB. The results of these studies provide for an in-depth understanding of the microbial community dynamics and the formation mechanisms of flavor compounds in the fermentation process of CFB.

## Figures and Tables

**Table 1:** Changes in organic acid contents during CFB fermentation.

Organic Acids	Chi-Flavor Baijiu (mg/L)	Fermented 15 d with *P. anomala* and *L. plantarum* (mg/L)
Acetic acid (A)	1402.25 ± 19.24 ^g^	1212.09 ± 31.79 ^h^
Malic acid (B)	1373.95 ± 9.99 ^g^	1585.81 ± 15.23 ^f^
Succinic acid (C)	1642.16 ± 49.12 ^f^	1854.89 ± 59.93 ^e^
Lactic acid (D)	2165.59 ± 45.58 ^c^	2081.14 ± 27.40 ^d^
Total organic acids	7582.95 ± 83.21 ^b^	8435.17 ± 76.30 ^a^

Values were shown as the mean ± SE (n = 3). Different letters within the same column indicate significant differences by Tukey-Kramer multiple comparison test (*p* < 0.05).

## Data Availability

The original contributions presented in the study are included in the article/Supplementary Material, further inquiries can be directed to the corresponding authors.

## Supplementary Materials

The following supporting information can be downloaded at: https://www.mdpi.com/article/10.3390/foods13213497/s1, Table S1: Orthogonal experimental design of *Pichia anomala*; Table S2: Orthogonal experimental design of *Lactobacillus plantarum*; Table S3: Orthogonal experimental design of mixed strains; Table S4: The variation in the contents of flavor substances in the fermentation process; Figure S1: The fermentation process of CFB; Figure S2: Changes in total organic acid content during CFB fermentation; Figure S3: Plots of principal component analysis and OPLS-DA model correlation analysis for volatile flavor substances; Figure S4: The chromatograms for analysis of standards and real samples.

## References

[1] Wang Q., Wang C., Xiang X., Xu H., Han G. (2022). Analysis of microbial diversity and succession during Xiaoqu Baijiu fermentation using high-throughput sequencing technology. Eng. Life Sci..

[2] Zhao D., Sun J., Sun B., Zhao M., Zheng F., Huang M., Sun X., Li H. (2017). Intracellular antioxidant effect of vanillin, 4-methylguaiacol and 4-ethylguaiacol: Three components in Chinese Baijiu. RSC Adv..

[3] Wu Z., Qin D., Duan J., Li H., Sun J., Huang M., Sun B. (2021). Characterization of benzenemethanethiol in sesame-flavour baijiu by high-performance liquid chromatography-mass spectrometry and sensory science. Food Chem..

[4] Li D., Jia F., Wang L., Chang F. (2023). The initial composition and structure of microbial community determined the yield and quality of Baijiu during the spontaneous fermentation. Int. Microbiol..

[5] Wang P., Wu Q., Jiang X., Wang Z., Tang J., Xu Y. (2017). Bacillus licheniformis affects the microbial community and metabolic profile in the spontaneous fermentation of Daqu starter for Chinese liquor making. Int. J. Food Microbiol..

[6] He G., Huang J., Wu C., Jin Y., Zhou R. (2019). Bioturbation effect of fortified Daqu on microbial community and flavor metabolite in Chinese strong-flavor liquor brewing microecosystem. Food Res. Int..

[7] Gao J., Qin J., Ye F., Ding F., Liu G., Li A., Ren C., Xu Y. (2022). Constructing simplified microbial consortia to improve the key flavour compounds during strong aroma-type Baijiu fermentation. Int. J. Food Microbiol..

[8] Qu C., Peng L., Fei Y., Liang J., Bai W., Liu G. (2023). Screening ester-producing yeasts to fortify the brewing of rice-flavor Baijiu for enhanced aromas. Bioengineered.

[9] Swangkeaw J., Vichitphan S., Butzke C., Vichitphan K. (2009). The characterisation of a novel *Pichia anomala* β-glucosidase with potentially aroma-enhancing capabilities in wine. Ann. Microbiol..

[10] Ye M., Yue T., Yuan Y. (2014). Effects of sequential mixed cultures of Wickerhamomyces anomalus and Sac-charomyces cerevisiae on apple cider fermentation. FEMS Yeast Res..

[11] Todorov S.D., Franco B.D.G.D.M. (2010). *Lactobacillus plantarum*: Characterization of the Species and Application in Food Production. Food Rev. Int..

[12] Urcan A.C., Criste A.D., Bobiș O., Cornea-Cipcigan M., Giurgiu A.-I., Dezmirean D.S. (2024). Evaluation of Functional Properties of Some Lactic Acid Bacteria Strains for Probiotic Applications in Apiculture. Microorganisms.

[13] Fu Z., Sun B., Li X., Fan G., Teng C., Alaa A., Jia Y. (2018). Isolation and characterization of a high ethyl acetate-producing yeast from Laobaigan *Daqu* and its fermentation conditions for producing high-quality *Baijiu*. Biotechnol. Biotechnol. Equip..

[14] Huang X., Fan Y., Lu T., Kang J., Pang X., Han B., Chen J. (2020). Composition and Metabolic Functions of the Microbiome in Fermented Grain during Light-Flavor *Baijiu* Fermentation. Microorganisms.

[15] Deng N., Du H., Xu Y. (2020). Cooperative Response of *Pichia kudriavzevii* and *Saccharomyces cerevisiae* to Lactic Acid Stress in Baijiu Fermentation. J. Agric. Food Chem..

[16] Fan G., Teng C., Xu D., Fu Z., Liu P., Wu Q., Yang R., Li X. (2019). Improving Ethyl Acetate Production in Baijiu Manufacture by *Wickerhamomyces anomalus* and *Saccharomyces cerevisiae* Mixed Culture Fermentations. BioMed Res. Int..

[17] Zhang W., He S.G., Han X.L., Li H., Yu J.X., Jiang W., Tang K.T. (2010). Analysis of Flavor Components of Soybean-flavor Liquor. Liquor.-Mak. Sci. Technol..

[18] Fan H., Fan W., Xu Y. (2015). Characterization of Key Odorants in Chinese Chixiang Aroma-Type Liquor by Gas Chromatography–Olfactometry, Quantitative Measurements, Aroma Recombination, and Omission Studies. J. Agric. Food Chem..

[19] Jia W., Fan Z., Du A., Li Y., Zhang R., Shi Q., Shi L., Chu X. (2020). Recent advances in Baijiu analysis by chromatography based technology—A review. Food Chem..

[20] Duppeti H., Kempaiah B.B., Manjabhatta S.N. (2022). Influence of processing conditions on the aroma profile of *Litopenaeus vannamei* by SPME-GC-MS. Flavour. Fragr. J..

[21] Wang J., Yan C., Ma C., Huang S., Chang X., Li Z., Chen X., Li X. (2023). Effects of two kinds of Bacillus on flavour formation of Baijiu solid-state fermentation with pure mixed bacteria. Int. J. Food Sci. Technol..

[22] Liu H., Sun B. (2018). Effect of Fermentation Processing on the Flavor of Baijiu. J. Agric. Food Chem..

[23] Zhao W., Liang Z., Qian M., Li X., Dong H., Bai W., Wei Y., He S. (2022). Evolution of microbial communities during fermentation of Chi-flavor type Baijiu as determined by high-throughput sequencing. LWT Food Sci. Technol..

[24] Liang Z., Lin X., He Z., Li W., Ren X., Lin X. (2020). Dynamic changes of total acid and bacterial communities during the traditional fermentation of Hong Qu glutinous rice wine. Electron. J. Biotechnol..

[25] Zhao W., Qian M., Dong H., Liu X., Bai W., Liu G., Lv X.-C. (2022). Effect of Hong Qu on the flavor and quality of Hakka yellow rice wine (Huangjiu) produced in Southern China. LWT Food Sci. Technol..

[26] (2022). Methods of Analysis for Chinese Baijiu.

[27] Huang T., Lu Z.-M., Peng M.-Y., Liu Z.-F., Chai L.-J., Zhang X.-J., Shi J.-S., Li Q., Xu Z.-H. (2021). Combined effects of fermentation starters and environmental factors on the microbial community assembly and flavor formation of Zhenjiang aromatic vinegar. Food Res. Int..

[28] Yang Y., Xia Y., Hu W., Tao L., Liu H., Xie C., Bai W., Ai L. (2020). Soaking induced discrepancies in oenological properties, flavor profiles, microbial community and sensory characteristic of Huangjiu (Chinese rice wine). LWT Food Sci. Technol..

[29] Chen L., Yan R., Zhao Y., Sun J., Zhang Y., Li H., Zhao D., Wang B., Ye X., Sun B. (2023). Characterization of the aroma release from retronasal cavity and flavor perception during baijiu consumption by Vocus-PTR-MS, GC×GC-MS, and TCATA analysis. LWT Food Sci. Technol..

[30] Qian M., Ruan F., Zhao W., Dong H., Bai W., Li X., Huang X., Li Y. (2023). The dynamics of physicochemical properties, microbial community, and flavor metabolites during the fermentation of semi-dry Hakka rice wine and traditional sweet rice wine. Food Chem..

[31] Fei Y., Wang Y., Zhang Z., Liang J., Zhao W., Bai W., He S., Liu Y. (2023). Effects of traditional starter and the Round-Koji-Maker starter on microbial communities and volatile flavours of Chi-Flavour Baijiu. Int. J. Food Sci. Technol..

[32] Jiang H., Ou S., Ye J., Qian M., Wen J., Qi H., Zeng X., Zhao W., Bai W. (2024). Variation of volatile flavor substances in salt-baked chicken during processing. Food Chem. X.

[33] Othman M., Ariff A.B., Rios-Solis L., Halim M. (2017). Extractive Fermentation of Lactic Acid in Lactic Acid Bacteria Cultivation: A Review. Front. Microbiol..

[34] Pang X., Chen C., Huang X., Yan Y., Chen J., Han B. (2021). Influence of indigenous lactic acid bacteria on the volatile flavor profile of light-flavor Baijiu. LWT Food Sci. Technol..

[35] Tiwari P., Upadhyay M. (2012). Characterization of a thermo tolerant lipase from *Pichia anomala*. Eur. J. Exp. Biol..

[36] Xu Y., Zhao J., Liu X., Zhang C., Zhao Z., Li X., Sun B. (2021). Flavor mystery of Chinese traditional fermented Baijiu: The great contribution of ester compounds. Food Chem..

[37] Wang G., Jing S., Wang X., Zheng F., Li H., Sun B., Li Z. (2022). Evaluation of the Perceptual Interaction among Ester Odorants and Nonvolatile Organic Acids in Baijiu by GC-MS, GC-O, Odor Threshold, and Sensory Analysis. J. Agric. Food Chem..

[38] Lane S., Turner T.L., Jin Y. (2023). Glucose assimilation rate determines the partition of flux at pyruvate between lactic acid and ethanol in *Saccharomyces cerevisiae*. Biotechnol. J..

[39] Zhao W., Liang M., Fan P., Pan L., Liang J., Fei Y., Bai W. (2024). Effect of hydrolyzed soybean on the volatile flavors and microbial community in the traditional brewing process of chi-flavor Baijiu. J. Food Sci..

[40] Yin X., Yoshizaki Y., Kurazono S., Sugimachi M., Takeuchi H., Han X.-L., Okutsu K., Futagami T., Tamaki H., Takamine K. (2020). Characterization of flavor compounds in rice-flavor baijiu, a traditional chinese distilled liquor, compared with Japanese distilled liquors, awamori and kome-shochu. Food Sci. Technol. Res..

[41] Richter H., Loftus S.E., Angenent L.T. (2013). Integrating syngas fermentation with the carboxylate platform and yeast fermentation to reduce medium cost and improve biofuel productivity. Environ. Technol..

[42] Liu S., Ma D., Li Z., Sun H., Mao J., Shi Y., Han X., Zhou Z., Mao J. (2021). Assimilable nitrogen reduces the higher alcohols content of huangjiu. Food Control.

[43] Chen G., Yuan Y., Tang S., Yang Z., Wu Q., Liang Z., Chen S., Li W., Lv X., Ni L. (2023). Comparative analysis of microbial communities and volatile flavor components in the brewing of Hongqu rice wines fermented with different starters. Curr. Res. Food Sci..

[44] Grosch W. (2001). Evaluation of the key odorants of foods by dilution experiments, aroma models and omission. Chem. Senses.

[45] Qian X., Lan Y., Han S., Liang N., Zhu B., Shi Y., Duan C. (2020). Comprehensive investigation of lactones and furanones in icewines and dry wines using gas chromatography-triple quadrupole mass spectrometry. Food Res. Int..

[46] (2018). Chi-Flavor Baijiu.

[47] Wang Y., Fu Y., Zhang Q., Zhu Y., Yang Q., Bian C., Zhao L.-L., Chen Q., Bi H.-J., Yang X.-H. (2024). Enhancement of ester biosynthesis in blueberry wines through co-fermentation via cell–cell contact between *Torulaspora delbrueckii* and *Saccharomyces cerevisiae*. Food Res. Int..

[48] Wang L., Zhang Y., Kiprowska M., Guo Y., Yamamoto K., Li X. (2021). Diethyl Succinate Modulates Microglial Polarization and Activation by Reducing Mitochondrial Fission and Cellular ROS. Metabolites.

[49] Styger G., Jacobson D., Bauer F.F. (2011). Identifying genes that impact on aroma profiles produced by *Saccharomyces cerevisiae* and the production of higher alcohols. Appl. Microbiol. Biotechnol..

[50] Hirst M., Richter C. (2016). Review of Aroma Formation through Metabolic Pathways of *Saccharomyces cerevisiae* in Beverage Fermentations. Am. J. Enol. Vitic..

[51] Zhao C., Yan X., Yang S., Chen F. (2017). Screening of Bacillus strains from Luzhou-flavor liquor making for high-yield ethyl hexanoate and low-yield propanol. LWT Food Sci. Technol..

[52] Yang F., Liu Y., Chen L., Li J., Wang L., Du G. (2020). Genome sequencing and flavor compound biosynthesis pathway analyses of *Bacillus licheniformis* isolated from Chinese *Maotai*-flavor liquor-brewing microbiome. Food Biotechnol..

[53] Alvarez-Macarie E., Baratti J. (2000). Short chain flavour ester synthesis by a new esterase from *Bacillus licheniformis*. J. Mol. Catal. B Enzym..

[54] Zeng X., Mo Z., Zheng J., Wei C., Dai Y., Yan Y., Qiu S. (2023). Effects of biofilm and co-culture with *Bacillus velezensis* on the synthesis of esters in the strong flavor Baijiu. Int. J. Food Microbiol..

[55] Lomascolo A., Lesage-Meessen L., Haon M., Navarro D., Antona C., Faulds C., Marcel A. (2001). Evaluation of the potential of *Aspergillus niger* species for the bioconversion of L-phenylalanine into 2-phenylethanol. World J. Microbiol. Biotechnol..

[56] Zhou R., Song Q., Xia H., Song N., Yang Q., Zhang X., Yao L., Yang S., Dai J., Chen X. (2023). Isolation and Identification of Non-Saccharomyces Yeast Producing 2-Phenylethanol and Study of the Ehrlich Pathway and Shikimate Pathway. J. Fungi.

[57] Karimi K., Zamani A. (2013). Mucor indicus: Biology and industrial application perspectives: A review. Biotechnol. Adv..

[58] Wang Y., Zhang Z., Lu X., Zong H., Zhuge B. (2020). Genetic engineering of an industrial yeast *Candida glycerinogenes* for efficient production of 2-phenylethanol. Appl. Microbiol. Biotechnol..

[59] Xu Y., Wu M., Niu J., Lin M., Zhu H., Wang K., Li X., Sun B. (2023). Characteristics and Correlation of the Microbial Communities and Flavor Compounds during the First Three Rounds of Fermentation in Chinese Sauce-Flavor Baijiu. Foods.

[60] Dellomonaco C., Clomburg J.M., Miller E.N., Gonzalez R. (2011). Engineered reversal of the β-oxidation cycle for the synthesis of fuels and chemicals. Nature.

[61] Zhuang X.-Y., Zhang Y.-H., Xiao A.-F., Zhang A.-H., Fang B.-S. (2022). Key Enzymes in Fatty Acid Synthesis Pathway for Bioactive Lipids Biosynthesis. Front. Nutr..

[62] Kruis A.J., Bohnenkamp A.C., Patinios C., van Nuland Y.M., Levisson M., Mars A.E., van den Berg C., Kengen S.W., Weusthuis R.A. (2019). Microbial production of short and medium chain esters: Enzymes, pathways, and applications. Biotechnol. Adv..

