# Supplementary material for: Effect of Mixed Strains on Microbial Community and Flavor Metabolites in Fermentation Process of Chi-Flavor Baijiu
Source: Foods. 2024 Oct 31;13(21):3497. doi: 10.3390/foods13213497 (PMC11545386; doi:10.3390/foods13213497)
Supplement: Supplementary file 1 [file foods-13-03497-s001.zip › foods-3183927-supplementary.pdf]

Table S1. Orthogonal experimental design of *Pichia anomala*.

Factor level	Factor			
	A Inoculation rate (%)	B **Fermentation Temperature** (℃)	C Initial pH	D **Fermentation Time** (d)
1	6	28	5	3
2	8	30	6	4
3	10	32	7	5

Table S2. Orthogonal experimental design of *Lactobacillus plantarum*.

Factor level	Factor			
	A Inoculation rate (%)	B **Fermentation Temperature** (℃)	C Initial pH	D **Fermentation Time**(d)
1	8	32	4	5
2	10	36	5	6
3	12	40	6	7

Table S3. Orthogonal experimental design of mixed strains.

Factor level	Factor			
	A Microbial ratio	B Inoculation rate (%)	C Initial pH	D **Fermentation Temperature** (℃)
1	5:5	8	6	32
2	7:3	10	7	34
3	9:1	12	8	36

Table S4. The variation in the content of flavor substances in the fermentation process.

Num.	Volatile flavor compounds	RI	Content (mg/L)															
			12h	1d	2d	4d	KB6d	KB7d	KB8d	KB9d	KB11d	KB15d	SY6d	SY7d	SY8d	SY9d	SY11d	SY15d
	**Alcohol**																	
A1	Ethane, 1,1-diethoxy-	861	1.272	1.397	1.442	1.116	ND	ND	ND	ND	ND	ND	ND	ND	ND	ND	ND	ND
A2	2-Methyl-1-propanol	1116	1.078	1.372	1.981	2.540	4.009	2.347	4.780	2.2640	1.987	2.316	1.437	0.953	1.570	1.836	6.130	7.567
A3	1-Butanol	1192	ND	ND	ND	ND	ND	ND	ND	ND	ND	ND	ND	0.246	0.063	ND	ND	ND
A4	3-Methyl-1-butanol	1210	28.474	27.066	31.268	27.140	30.188	16.267	36.389	12.127	9.764	12.445	26.727	18.364	21.221	35.664	47.848	85.708
A5	1-Hexanol	1357	0.597	0.750	0.815	0.539	0.617	0.814	0.972	0.571	0.504	0.474	0.717	0.809	1.046	1.408	1.169	0.731
A6	1-Propanol, 3-ethoxy-	1378	0.116	0.218	0.239	0.174	0.273	0.416	0.391	0.148	0.130	0.240	0.318	0.544	0.312	0.340	0.177	0.321
A7	4-Hexen-1-ol, (E)-	1413	0.046	0.078	0.095	0.087	0.040	0.076	0.089	0.078	0.101	0.109	0.068	0.048	0.056	0.084	0.107	0.063
A8	1-Heptanol	1454	ND	ND	ND	ND	ND	ND	0.292	0.060	0.027	ND	ND	0.088	0.022	0.023	0.114	ND
A9	1-Octanol	1556	0.078	0.137	0.656	0.448	0.497	1.776	1.207	0.085	0.085	0.118	0.972	0.835	0.251	0.352	0.381	0.657
A10	2,3-Butanediol	1576	0.049	0.077	0.098	0.105	0.093	0.082	0.082	0.147	0.172	0.571	0.148	0.075	0.107	0.103	0.114	0.401
A11	Tricosan-2-ol	1651	0.127	0.104	0.123	0.137	ND	ND	ND	ND	ND	ND	0.146	ND	ND	ND	ND	ND
A12	2-Furanmethanol	1665	ND	ND	ND	ND	ND	ND	ND	ND	ND	ND	ND	ND	ND	ND	ND	0.135
A13	Benzyl alcohol	1867	33.866	21.358	11.897	10.541	12.447	32.955	27.513	1.942	1.596	2.835	12.606	7.444	1.204	1.544	8.608	14.751
A14	Phenylethyl alcohol	1904	56.399	65.002	59.068	49.381	50.387	127.133	115.93	39.161	36.179	13.252	48.195	31.593	35.985	37.839	36.982	65.735
A15	3-Phenylpropanol	2034	2.027	0.607	0.486	0.048	0.471	1.418	0.884	0.595	0.476	0.175	0.068	0.056	0.037	0.047	0.278	1.139
A16	Spathulenol	2111	0.009	0.013	0.016	0.021	ND	ND	ND	ND	0.096	0.038	0.017	0.016	0.014	0.012	0.119	0.680
A17	2-Methoxybenzyl	2155	0.785	0.413	0.361	0.389	0.426	1.503	1.151	0.784	0.580	0.402	0.582	0.345	0.421	0.462	0.574	0.632

	alcohol																	
A18	Benzenepropanol, 2-methoxy-	2317	0.4621	0.4056	0.210	0.194	ND	ND	ND	0.008	0.013	0.055	0.086	0.057	0.057	0.050	0.035	0.027
	**Total Alcohols**		125.392±3.961c	119.005±1.038d	108.762±1.423e	92.866±1.775g	92.093±0.256g	61.480±0.357i	62.372 ±0.640i	79.770 ±1.285h	102.643 ±0.579f	178.554 ±2.811b	99.453 ±1.642f	184.792 ±4.567a	189.692 ±7.061a	57.976 ±1.177i	51.716 ±0.991j	33.036 ±0.722k
	**Esters**																	
B1	Ethyl acetate	837	ND	0.546	7.231	9.727	10.859	22.915	33.969	30.486	23.392	13.505	18.804	35.939	40.010	63.817	43.720	34.812
B2	Propanoic acid, ethyl ester	1002	ND	ND	ND	ND	ND	0.639	0.694	0.105	0.018	0.020	ND	ND	0.057	0.078	0.145	0.705
B3	Isoamyl acetae	1121	0.114	0.217	0.156	0.235	0.270	0.281	0.191	0.231	0.440	0.331	0.250	0.212	0.207	0.192	0.148	0.186
B4	Ethyl hexanoic	1232	0.026	0.248	0.241	0.238	0.314	0.341	0.401	0.210	0.147	0.145	0.373	0.397	0.451	0.236	0.198	0.173
B5	Phenacyl thiocyanate	1290	1.579	3.966	4.220	6.682	15.585	ND	ND	ND	ND	9.686	5.380	2.597	2.191	3.617	3.722	3.763
B6	Ethyl lactate	1343	ND	ND	ND	0.584	ND	ND	ND	6.615	15.293	10.952	0.748	5.830	7.637	8.747	8.532	15.858
B7	2-Ethoxyphenyl isothiocyanate	1400	ND	ND	ND	ND	4.177	9.889	14.641	2.070	2.505	2.709	6.925	1.681	1.086	1.408	8.178	12.360
B8	Ethyl caprylate	1429	0.571	0.563	1.071	1.488	1.160	2.003	1.300	1.352	1.800	2.131	1.906	2.297	3.469	2.809	3.042	3.311
B9	2,4-Hexadienoic acid, ethyl ester	1502	2.582	1.478	1.939	0.109	0.741	2.810	0.858	0.044	0.090	0.085	0.132	0.227	0.026	0.057	0.121	1.061
B10	Formic acid, octyl ester	1551	1.228	ND	ND	0.107	ND	ND	ND	ND	ND	ND	ND	0.376	ND	ND	ND	0.114
B11	Propanoic acid, 2-hydroxy-pentylester	1562	ND	ND	ND	ND	ND	ND	ND	ND	0.215	0.337	ND	ND	ND	ND	ND	ND
B12	Ethyl caprate	1643	0.489	0.579	0.527	0.232	0.376	1.173	0.479	0.587	0.862	1.251	0.752	1.477	1.264	1.360	1.946	1.582
B13	Ethyl benzoate	1659	0.412	0.263	0.743	0.635	0.916	2.788	2.423	1.347	1.282	2.064	0.950	3.728	4.113	2.999	2.568	2.533

B14	Diethyl succinate	1678	ND	ND	ND	ND	ND	ND	ND	ND	ND	1.526	ND	ND	ND	ND	4.771	9.897
B15	Acetic acid, phenylmethyl ester	1722	1.350	0.189	0.208	0.776	0.497	0.243	ND	ND	ND	ND	ND	ND	ND	ND	ND	0.996
B16	Crystal violet lactone	1745	ND	0.037	0.070	0.406	ND	ND	ND	ND	ND	ND	ND	ND	ND	ND	ND	0.352
B17	Benzeneacetic acid, ethyl ester	1770	ND	ND	ND	ND	ND	0.602	0.400	0.289	0.267	0.138	ND	ND	ND	ND	ND	ND
B18	4-Ethylbenzoic ac id, pentyl ester	1779	ND	0.013	0.018	0.089	ND	ND	ND	ND	ND	ND	ND	ND	0.026	ND	ND	ND
B19	Phenethyl acetate	1803	2.324	1.907	1.107	1.110	1.720	2.320	1.697	1.615	1.269	1.203	1.802	2.497	2.578	2.278	2.267	2.216
B20	Ethyl laurate	1840	0.201	0.140	0.157	0.208	0.398	0.456	0.126	0.094	0.068	0.066	0.216	0.199	0.203	0.259	0.265	0.802
B21	γ-Nonanolactone	2008	0.062	0.159	0.167	0.170	0.148	0.596	0.377	0.235	0.202	0.149	0.415	0.889	0.150	0.194	0.198	0.555
B22	Tetradecanoic acid, ethyl ester	2043	ND	ND	ND	0.522	0.564	3.167	1.351	1.104	1.071	0.554	0.623	0.716	0.752	1.150	0.874	1.933
B23	Diethyl azelate	2209	ND	ND	ND	ND	ND	ND	ND	ND	ND	ND	ND	ND	ND	ND	ND	0.406
B24	Benzeneacetic acid, 2-propenyl ester	2244	ND	ND	ND	0.035	ND	ND	0.505	0.038	ND	0.097	0.143	ND	ND	ND	ND	1.583
	**Total Esters**		13.994± 0.423m	14.378± 0.289m	22.585± 0.636l	27.168± 0.720k	46.504± 0.491i	69.992± 1.775e	75.670 ±2.036d	103.659 ±2.650b	97.489 ±2.581c	137.195 ±1.930a	41.263 ±0.973j	63.365 ±0.620f	66.288 ±1.439f	52.419 ±1.068gh	53.920 ±2.889g	49.509 ±1.117hi
	**Aldehydes**																	
C1	Octanal	1281	ND	ND	ND	ND	ND	ND	ND	ND	ND	ND	ND	ND	0.027	0.040	ND	ND
C2	Nonanal	1389	0.487	0.418	0.401	0.386	0.379	0.742	0.382	0.084	0.051	0.036	0.036	ND	ND	0.048	0.058	ND
C3	(E)-2-Octenal	1419	8.350	2.873	1.392	1.010	1.525	2.090	2.401	0.118	0.109	0.205	0.769	0.229	0.102	0.062	0.705	0.748
C4	Furfural	1451	ND	ND	ND	ND	ND	ND	ND	0.062	0.069	0.187	ND	ND	ND	0.129	0.349	1.072

C5	Decanal	1492	0.109	0.185	0.340	0.108	0.107	0.037	ND	ND	ND	0.060	0.018	ND	ND	ND	ND	ND
C6	Benzaldehyde	1508	14.246	8.562	3.515	2.533	2.510	5.186	6.027	0.393	0.352	0.727	0.229	0.152	0.179	0.189	1.600	6.029
C7	Benzaldehyde, 2-methyl-	1628	1.004	0.769	0.341	ND	ND	ND	0.284	0.169	0.035	ND	ND	ND	ND	ND	ND	ND
C8	Benzeneacetaldehyde	1630	ND	ND	ND	ND	ND	ND	ND	ND	0.070	0.056	ND	ND	ND	ND	ND	0.248
C9	Benzaldehyde, 4-ethyl-	1690	0.323	0.045	0.047	0.065	0.130	0.138	ND	ND	ND	ND	ND	ND	ND	ND	ND	ND
C10	Benzaldehyde, 3,5-dimethyl-	1789	2.168	0.949	0.527	0.394	0.470	0.757	0.609	0.080	0.054	0.088	0.056	ND	ND	ND	0.332	0.850
C11	2-Propenal, 3-phenyl-	1813	ND	ND	ND	ND	0.051	0.064	ND	ND	ND	ND	ND	ND	ND	ND	ND	ND
C11	Benzaldehyde, 2-methoxy-	1949	0.055	ND	ND	ND	ND	ND	ND	ND	ND	ND	ND	ND	ND	ND	ND	0.153
C13	Cinnamaldehyde, (E)-	2015	ND	ND	ND	0.046	0.072	0.026	0.012	0.009	0.007	0.041	0.016	0.067	0.003	0.011	0.027	0.039
	**Total Aldehydes**		26.742± 0.369a	13.801 ±0.081b	6.564 ±0.191e	4.542 ±0.128g	1.124 ±0.042ij	0.448 ±0.016lm	0.311 ±0.014m	0.478 ±0.012lm	3.070 ±0.054h	9.139 ±0.210d	5.243 ±0.139f	9.040 ±0.075d	9.715 ±0.394c	0.916 ±0.041jk	0.747 ±0.015kl	1.436 ±0.030i
	**Acids**																	
D1	Propanoic acid	1532	0.124	0.014	0.016	0.025	0.030	0.036	0.132	0.033	0.022	0.024	0.016	0.031	0.026	0.027	0.100	0.273
D2	Propanoic acid, 2-methyl-	1567	ND	0.027	0.039	0.057	ND	ND	ND	0.077	0.024	ND	ND	0.325	0.054	0.098	0.156	0.503
D3	Butanoic acid	1636	ND	ND	ND	0.013	ND	ND	ND	ND	ND	ND	ND	0.010	0.007	ND	ND	ND
D4	Butanoic acid, 2-methyl-	1676	ND	0.050	0.077	0.028	ND	0.209	0.220	0.096	0.078	0.036	0.019	ND	ND	ND	0.151	0.093
D5	Benzoic acid, 2-	1783	ND	0.868	ND	ND	1.533	1.849	2.145	1.448	1.250	1.203	ND	1.857	1.727	0.007	0.084	0.657

	amino-4-methyl-																	
D6	Hexanoic acid	1845	0.714	0.249	0.358	0.393	0.244	0.260	0.376	0.350	0.169	0.119	0.477	0.449	0.317	0.300	0.139	0.243
D7	Octanoic acid	2059	1.398	0.310	0.154	0.123	0.128	0.127	0.148	0.013	0.014	0.023	0.013	0.010	0.008	0.006	0.071	0.252
D8	Nonanoic acid	2173	0.232	ND	ND	ND	ND	ND	ND	ND	ND	ND	ND	ND	ND	ND	ND	0.045
D9	n-Decanoic acid	2278	0.390	0.011	0.008	0.006	ND	0.118	0.027	0.013	0.009	0.329	ND	1.050	0.137	0.099	0.068	0.037
D10	Benzoic acid	2349	1.294	1.103	2.168	2.439	2.091	1.742	1.284	1.200	0.845	1.191	2.512	1.251	1.593	1.716	1.844	2.081
	**Total Acids**		4.153 ±0.140cd	2.632 ±0.102	2.821 ±0.046	3.084 ±0.085	3.035 ±0.028	4.982 ±0.082a	3.868 ±0.254e	2.253 ±0.078	2.613 ±0.140	4.185 ±0.050cd	4.027 ±0.021de	4.341 ±0.041b	4.332 ±0.130b	3.229 ±0.048	2.411 ±0.171	2.925 ±0.034
	**Phenols**																	
E1	Phenol	2011	ND	ND	0.067	0.046	ND	ND	ND	ND	ND	ND	ND	ND	ND	ND	ND	0.084
E2	2,4-Di-tert-butylphenol	2311	0.247	0.183	0.108	0.128	0.201	0.281	0.105	0.064	0.037	0.014	0.151	0.134	0.139	0.136	0.143	0.164
	**Total Phenols**		0.247 ±0.010b	0.183 ±0.009d	0.175 ±0.008d	0.175 ±0.011d	0.151 ±0.005e	0.134 ±0.004f	0.139 ±0.002ef	0.136 ±0.001f	0.144 ±0.005ef	0.247 ±0.009b	0.201 ±0.006c	0.281 ±0.009a	0.105 ±0.003g	0.064 ±0.002h	0.037 ±0.002i	0.014 ±0.001j
	**Hydrocarbons**																	
F1	Limonene	1186	ND	0.007	ND	ND	ND	ND	ND	0.010	0.004	ND	ND	ND	0.015	0.013	0.015	ND
F2	Styrene	1250	1.129	0.449	0.351	0.304	1.282	3.397	0.895	0.099	0.053	0.033	0.171	0.463	0.130	0.302	0.323	0.289
F3	Tetradecane	1394	ND	ND	ND	ND	ND	ND	ND	ND	0.004	ND	ND	ND	0.026	0.025	0.019	ND
F4	1,4-Hexadiene	1426	ND	0.020	0.080	0.119	0.222	0.187	0.080	0.024	0.023	0.016	ND	ND	ND	ND	ND	ND
F5	Cyclopentane, 1,3-dimethyl-, trans	1464	ND	0.016	0.018	0.020	ND	ND	ND	ND	ND	ND	0.016	ND	ND	ND	ND	ND
F6	Caryophyllene	1573	ND	ND	ND	ND	ND	ND	ND	ND	ND	ND	ND	ND	0.034	0.037	ND	ND
	**Total Hydrocarbons**		1.129 ±0.024c	0.492 ±0.014e	0.449 ±0.005g	0.442 ±0.006g	0.187 ±0.004j	0.463 ±0.018f	0.204 ±0.007j	0.377 ±0.008h	0.357 ±0.015h	0.289 ±0.005i	1.504 ±0.026b	3.584 ±0.023a	0.974 ±0.01d	0.133 ±0.007k	0.084 ±0.002l	0.049 ±0.004e
	**Ketones**																	
G1	2,2-	1285	0.886	4.024	3.645	7.726	15.490	14.577	11.436	10.132	9.365	12.434	6.700	4.093	4.502	3.702	4.852	5.645

	Dichlorobenzophenone																
G2	1-Octen-3-one	1297	5.700	1.573	0.825	0.146	1.803	2.565	2.709	0.129	0.152	0.334	0.055	0.098	0.165	0.104	0.230	0.552
G3	Ethanone, 1-(4-ethylphenyl)-	1485	ND	ND	ND	ND	ND	ND	ND	ND	0.023	0.018	0.005	0.027	0.030	0.034	0.059	0.041
G4	Ethanone, 1-(2-methylphenyl)-	1633	ND	ND	ND	ND	ND	1.180	0.086	ND	ND	ND	0.649	0.570	0.458	0.437	0.282	1.063
G5	Acetophenone	1639	0.620	ND	ND	ND	ND	0.416	0.348	0.112	0.090	0.051	ND	ND	ND	ND	ND	ND
	**Total Ketones**		7.206 ±0.024h	5.596 ±0.114i	4.470 ±0.085kl	7.872 ±0.166g	7.409 ±0.143h	4.788 ±0.048jk	5.155 ±0.051ij	4.278 ±0.062l	5.423 ±0.122ij	7.300 ±0.021h	17.292 ±0.295b	18.738 ±0.281a	14.578 ±0.285c	10.373 ±0.444e	9.630 ±0.316f	12.838 ±0.276d
	**Others**																
H1	Benzene, 1,2,4,5-tetramethyl-	1408	ND	ND	ND	ND	ND	ND	ND	ND	ND	ND	ND	ND	0.007	0.009	ND	ND
H2	Benzene, 1-ethenyl-4-ethyl-	1435	0.119	ND	ND	ND	ND	0.175	0.214	0.095	0.017	0.012	ND	0.025	ND	ND	ND	0.098
H3	2-Mercapto-4-phenylthiazole	1514	ND	0.355	0.822	0.413	1.932	2.194	4.089	1.354	0.516	0.795	0.613	2.244	ND	ND	ND	ND
H4	Ethanol, 2-(2-ethoxyethoxy)-	1623	ND	0.013	0.121	0.174	0.188	0.210	0.284	0.013	0.014	0.016	0.237	0.186	0.094	0.009	0.019	0.101
H5	Naphthalene	1715	0.608	0.360	0.573	0.648	0.791	0.868	0.592	0.430	0.385	0.379	1.920	1.351	0.732	0.482	0.166	0.350
H6	1H-Indole, 5-methyl-2-phenyl-	1878	0.051	0.039	0.164	0.019	0.100	0.037	0.019	0.018	0.031	0.016	0.008	0.080	0.076	0.028	0.025	0.016
H7	Naphthalene, 1,6-dimethyl-4-(1-methylethyl)-	2196	0.041	0.086	0.110	0.086	ND	ND	ND	ND	ND	ND	ND	ND	ND	ND	0.016	0.025
H8	Heptaethylene	2263	ND	ND	0.218	0.316	0.387	0.355	0.109	0.008	ND	ND	0.403	0.207	0.152	0.102	0.063	0.006

	glycol monomethyl ether																	
H9	15-Crown-5	2542	ND	ND	ND	ND	0.176	0.217	0.288	0.085	0.030	0.016	ND	ND	ND	ND	ND	ND
	**Others**		0.819 ±0.017j	0.853 ±0.015ij	2.007 ±0.211e	1.655 ±0.037f	3.180 ±0.047d	4.093 ±0.045b	1.061 ±0.031h	0.630 ±0.021k	0.288 ±0.028l	0.596 ±0.016k	3.574 ±0.03c	4.056 ±0.091b	5.594 ±0.051a	2.003 ±0.028e	0.993 ±0.051hi	1.234 ±0.093g

RI: retention index; ND: no detection; Values were shown as the mean ± SE (n = 3). Different letters within the same column indicate significant differences by Tukey-Kramer multiple comparison test ($p < 0.05$).

Figure S1. **The fermentation process of CFB.**

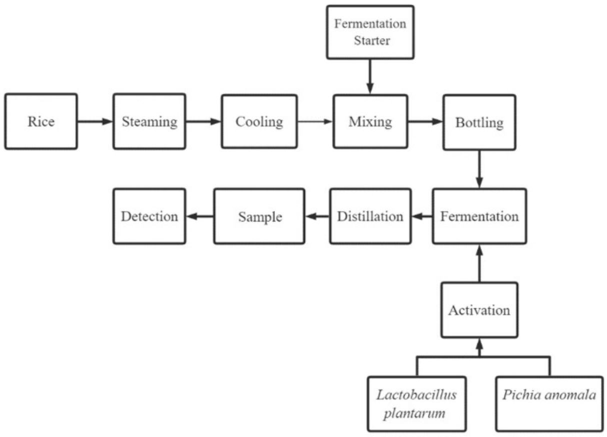

Figure S2 **Changes in total organic acid content during CFB fermentation.**

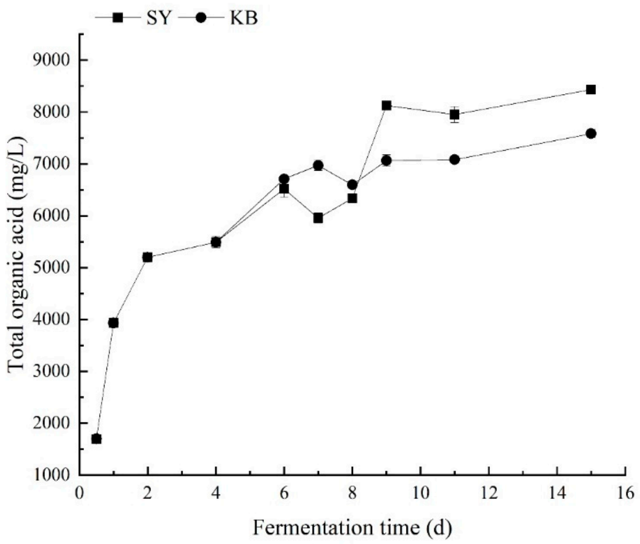

Figure S3 Plots of principal component analysis and OPLS-DA model correlation analysis for volatile flavor substances. (a. PCA-based analysis of volatile flavour substances in SY and KB groups, b. Principal component score plot; c. S-load plot based on OPLS-DA model analysis, d. VIP predicted value distribution plot based on OPLS-DA model analysis. Note: Red is the volatile flavor substances with VIP value > 1).

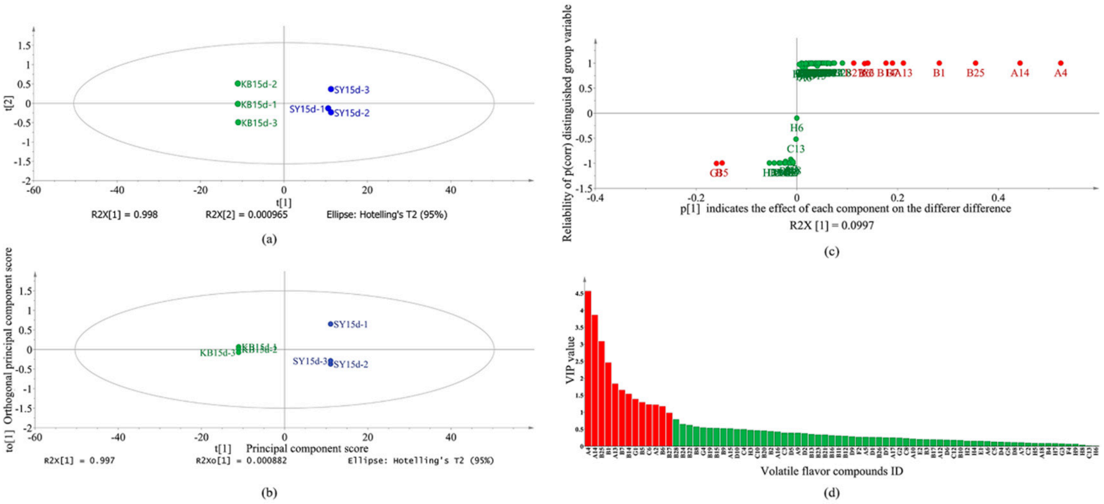

Figure S4 The chromatograms for standards and real samples analysis. (a. The chromatograms for standards; b. The chromatograms for samples (fermentation for 2 days), c. The chromatograms for samples (fermentation for 6 days; c_1 (SY); c_2 (KB)) d. The chromatograms for samples (fermentation for 9 days; d_1 (SY); d_2 (KB)) e. The chromatograms for samples (fermentation for 15 days; e_1 (SY); e_2 (KB)).

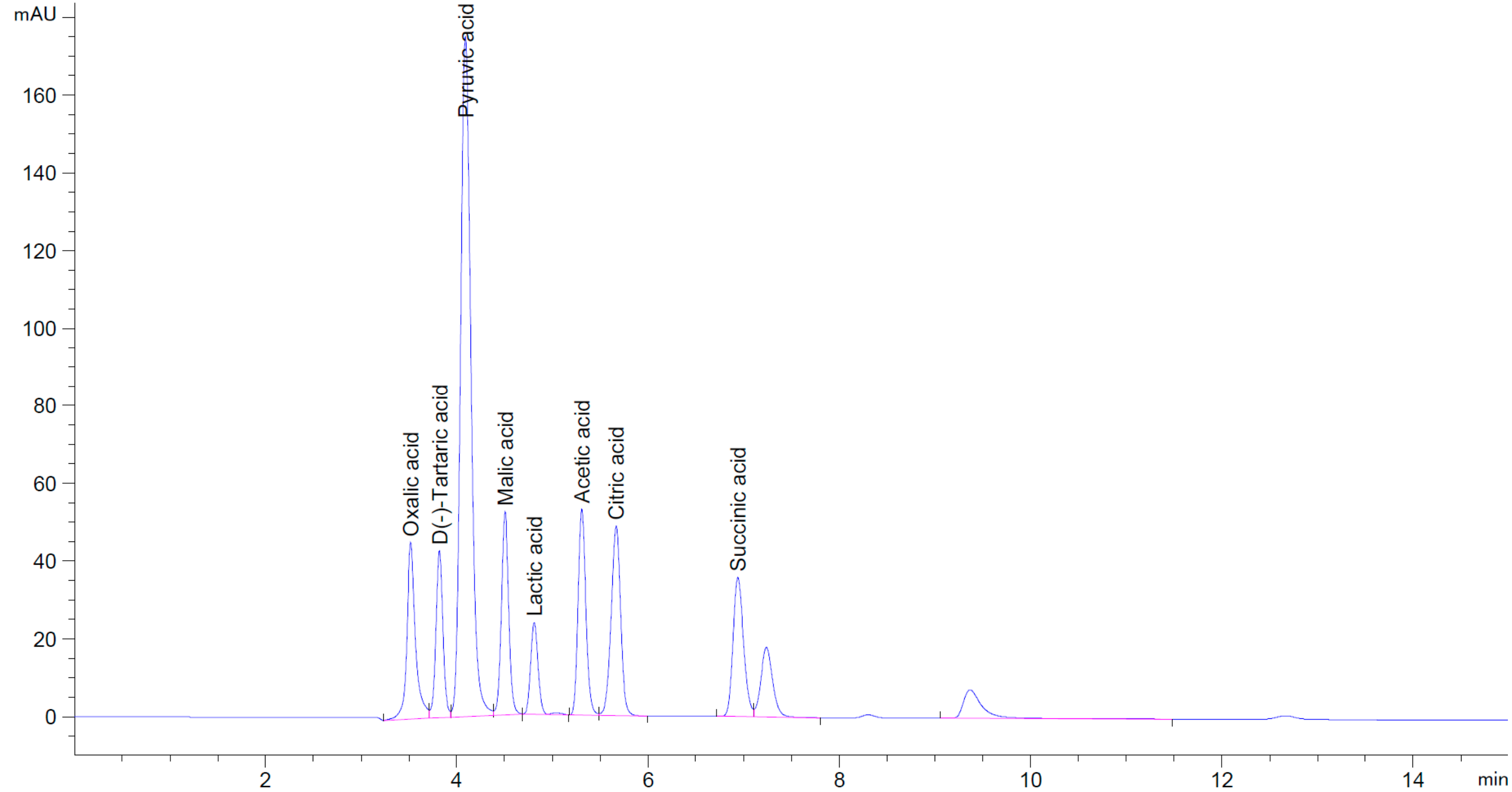

(a)

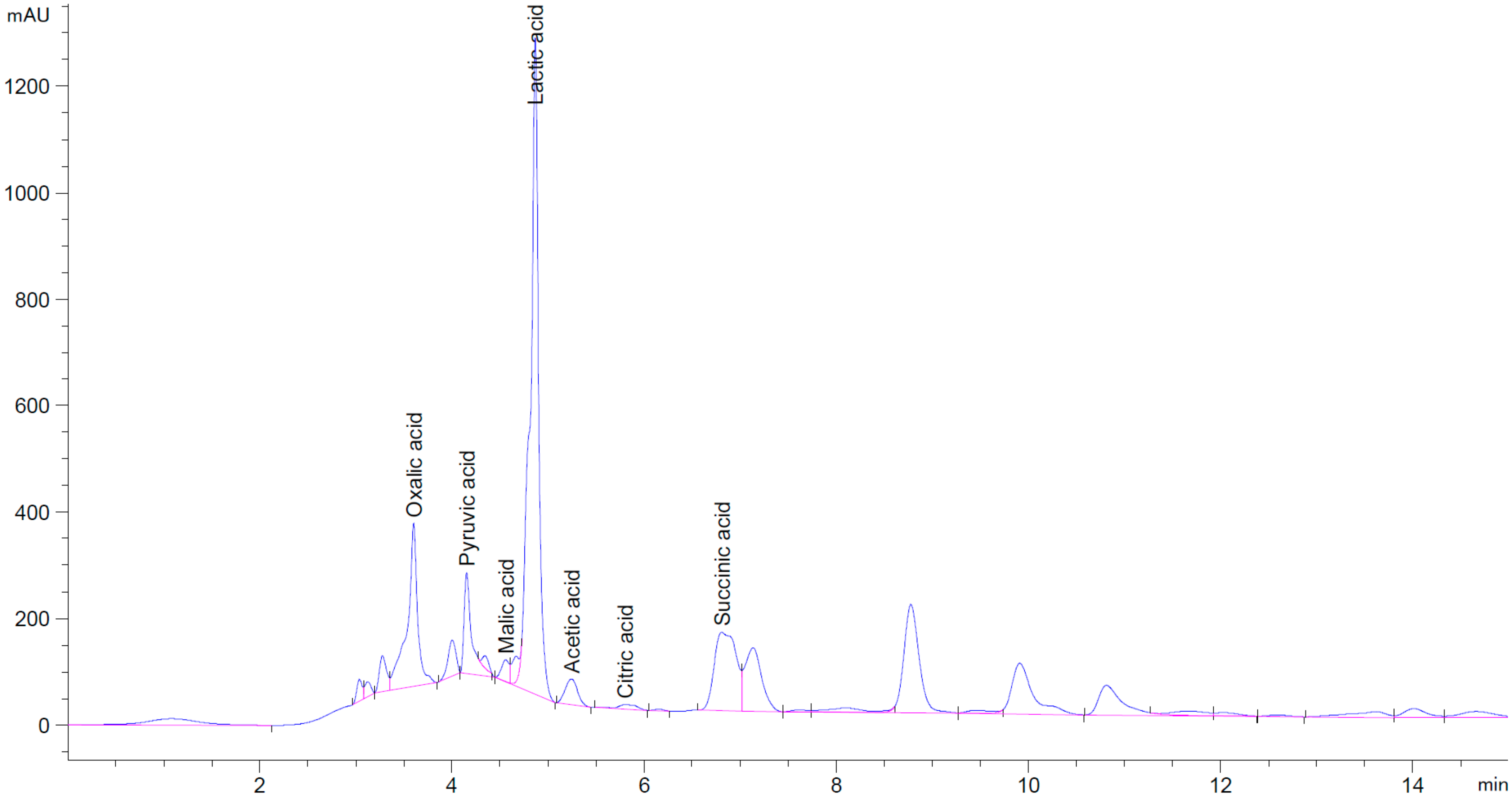
mAU
1200
1000
800
600
400
200
0
2
4
6
8
10
12
14
min
Oxalic acid
Pyruvic acid
Malic acid
Lactic acid
Acetic acid
Citric acid
Succinic acid

(b)

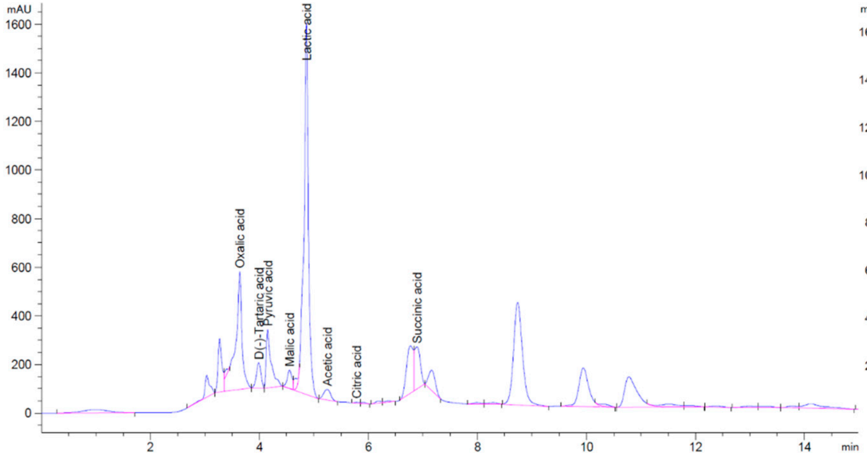
mAU
1600
1400
1200
1000
800
600
400
200
0
2
4
6
8
10
12
14
min
Oxalic acid
D(-)-Tartaric acid
Pyruvic acid
Malic acid
Lactic acid
Acetic acid
Citric acid
Succinic acid

(c$_1$)

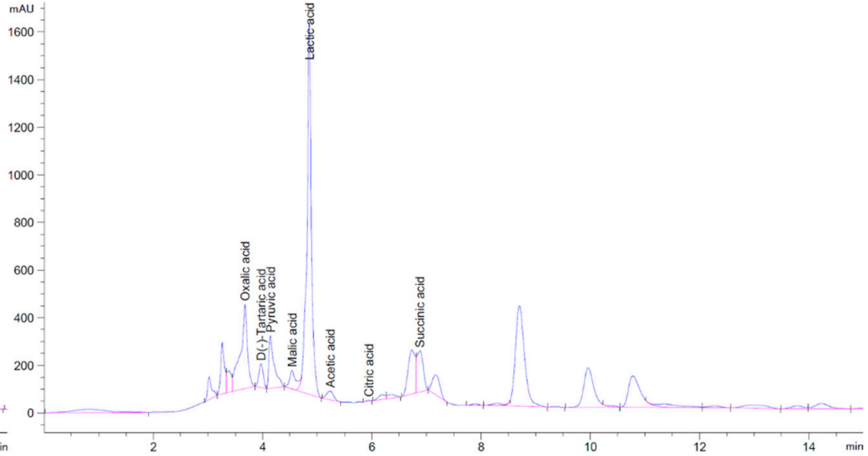
mAU
1600
1400
1200
1000
800
600
400
200
0
2
4
6
8
10
12
14
min
Oxalic acid
D(-)-Tartaric acid
Pyruvic acid
Malic acid
Lactic acid
Acetic acid
Citric acid
Succinic acid

(c$_2$)

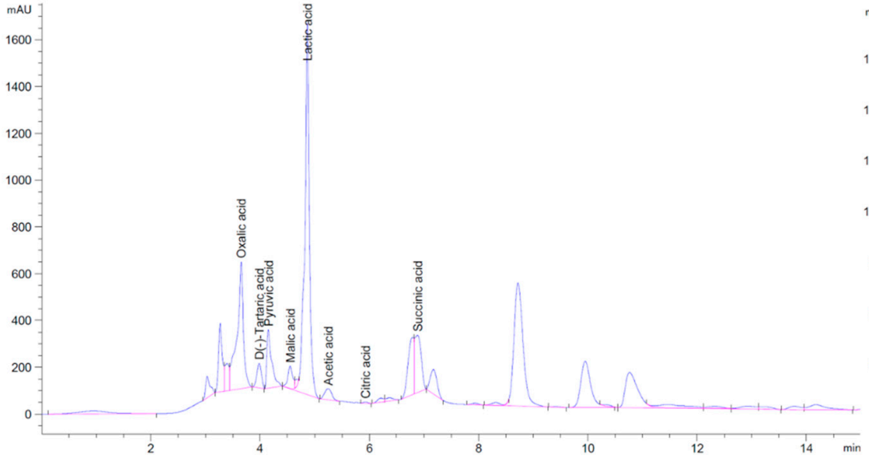
mAU
1600
1400
1200
1000
800
600
400
200
0
2
4
6
8
10
12
14
min
Oxalic acid
D(-)-Tartaric acid
Pyruvic acid
Malic acid
Lactic acid
Acetic acid
Citric acid
Succinic acid

(d$_1$)

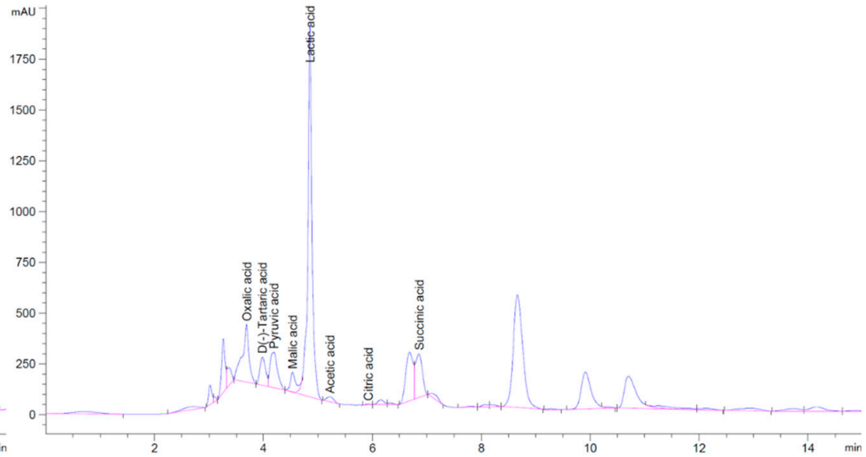
mAU
1750
1500
1250
1000
750
500
250
0
2
4
6
8
10
12
14
min
Oxalic acid
D(-)-Tartaric acid
Pyruvic acid
Malic acid
Lactic acid
Acetic acid
Citric acid
Succinic acid

(d$_2$)

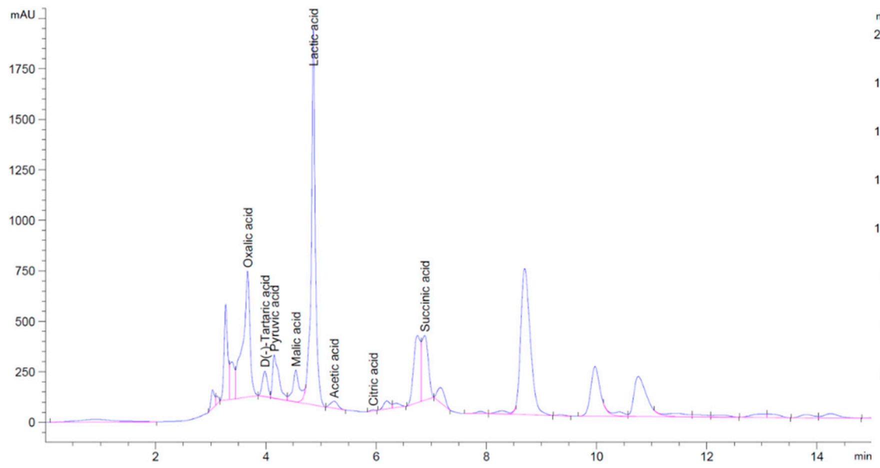

mAU
1750
1500
1250
1000
750
500
250
0
Oxalic acid
D(-)-Tartaric acid
Pyruvic acid
Malic acid
Lactic acid
Acetic acid
Citric acid
Succinic acid
2
4
6
8
10
12
14
min

(e$_1$)

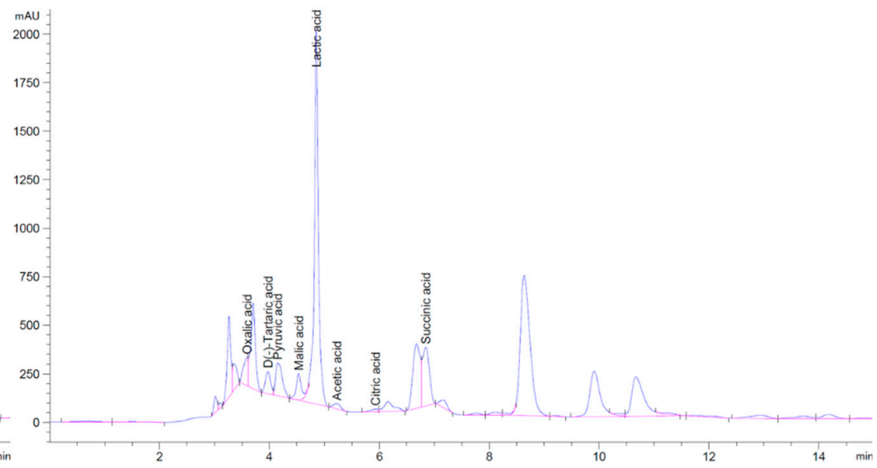

mAU
2000
1750
1500
1250
1000
750
500
250
0
Oxalic acid
D(-)-Tartaric acid
Pyruvic acid
Malic acid
Lactic acid
Acetic acid
Citric acid
Succinic acid
2
4
6
8
10
12
14
min

(e$_2$)
